# Developing Recombinant Antibodies by Phage Display Against Infectious Diseases and Toxins for Diagnostics and Therapy

**DOI:** 10.3389/fcimb.2021.697876

**Published:** 2021-07-07

**Authors:** Kristian Daniel Ralph Roth, Esther Veronika Wenzel, Maximilian Ruschig, Stephan Steinke, Nora Langreder, Philip Alexander Heine, Kai-Thomas Schneider, Rico Ballmann, Viola Fühner, Philipp Kuhn, Thomas Schirrmann, André Frenzel, Stefan Dübel, Maren Schubert, Gustavo Marçal Schmidt Garcia Moreira, Federico Bertoglio, Giulio Russo, Michael Hust

**Affiliations:** ^1^ Institut für Biochemie, Biotechnologie und Bioinformatik, Abteilung Biotechnologie, Technische Universität Braunschweig, Braunschweig, Germany; ^2^ Abcalis GmbH, Braunschweig, Germany; ^3^ YUMAB GmbH, Braunschweig, Germany

**Keywords:** phage display, infectious disease, recombinant antibodies, scFv antibodies, VHH antibodies

## Abstract

Antibodies are essential molecules for diagnosis and treatment of diseases caused by pathogens and their toxins. Antibodies were integrated in our medical repertoire against infectious diseases more than hundred years ago by using animal sera to treat tetanus and diphtheria. In these days, most developed therapeutic antibodies target cancer or autoimmune diseases. The COVID-19 pandemic was a reminder about the importance of antibodies for therapy against infectious diseases. While monoclonal antibodies could be generated by hybridoma technology since the 70ies of the former century, nowadays antibody phage display, among other display technologies, is robustly established to discover new human monoclonal antibodies. Phage display is an *in vitro* technology which confers the potential for generating antibodies from universal libraries against any conceivable molecule of sufficient size and omits the limitations of the immune systems. If convalescent patients or immunized/infected animals are available, it is possible to construct immune phage display libraries to select *in vivo* affinity-matured antibodies. A further advantage is the availability of the DNA sequence encoding the phage displayed antibody fragment, which is packaged in the phage particles. Therefore, the selected antibody fragments can be rapidly further engineered in any needed antibody format according to the requirements of the final application. In this review, we present an overview of phage display derived recombinant antibodies against bacterial, viral and eukaryotic pathogens, as well as microbial toxins, intended for diagnostic and therapeutic applications.

## Introduction

Antibodies are indispensable tools for basic research (Colwill and Gräslund, 2011), diagnostics (Kourea and Kotoula, 2016) and therapy (Kaplon and Reichert, 2021). In the past - and still today - polyclonal antibodies (blood serum) are produced in animals like horses (von Behring and Kitasato, 1890). The hybridoma technology was a milestone in antibody generation because it allowed the production of monoclonal antibodies (Köhler and Milstein, 1975). Despite these antibodies are monoclonal, cell secreted antibodies are not always monospecific, as unveiled by the results of a multicentric study on the detrimental effects of genetic diversity in individual hybridoma clones (Bradbury et al., 2018). Of 185 randomly selected hybridomas analyzed in this study, almost one third was found to contain additional productive heavy or light chains, resulting in antibodies with reduced affinity and more than one specificity. Other limitations of the hybridoma technology are the possible instability of the tetraploid, but also often aneuploid hybridoma cell lines (Pauza et al., 1993), the restriction of the immune system which prevents the generation of antibodies against highly conserved or self-antigens and most important the limitations to directly generate human antibodies (Winter and Milstein, 1991). The hybridoma technology most commonly provides murine antibodies for use in research and diagnostics. But, the therapeutic applications of murine antibodies are very limited because repeated administration of murine antibodies can cause a human anti-mouse antibody reaction (HAMA), which can lead also to severe side effects such as anaphylactic shock (Courtenay-Luck et al., 1986). While various humanization strategies are available to reduce the immunogenicity, these approaches are often laborious (Almagro and Fransson, 2008; Lo K.-M. et al., 2014). The human hybridoma technology allows the generation of human antibodies, (Cole et al., 1984; Hammond, 2010), but this approach will not be able to deliver antibodies against any targets because specific B-cells from humans would be needed and this is limited by ethical constraints. An alternative for human antibody generation is the use of transgenic animals in which the animal antibody gene repertoire is substituted with the corresponding human gene repertoire, but also this technology is limited by the restrictions of the immune system. This approach was successfully used for several approved therapeutic antibodies (Jakobovits, 1995; Lonberg and Huszar, 1995; Fishwild et al., 1996; Nelson et al., 2010).

A technology which allows to generate fully human antibodies right away, hence avoiding the side effects originating from HAMA, is antibody phage display. Because this approach uses an *in vitro* selection process, it does not have to rely on immunization and associated limitations, and can use entirely human gene repertoires. Phage display is based on the work of Georg P. Smith on filamentous phage, which infects *E. coli* and it is the most commonly used antibody display approach nowadays (Smith, 1985). Referring to the pan and the method of gold diggers, the antibody selection process was called “panning” (Parmley and Smith, 1988).

Around 1990/91, the M13 phage display technology was further developed for human antibody generation in parallel at three research sites: DKFZ in Heidelberg (Germany), MRC Laboratory of Molecular Biology in Cambridge (UK) and at the Scripps Research Institute in La Jolla (USA) (McCafferty et al., 1990; Barbas et al., 1991; Breitling et al., 1991; Clackson et al., 1991). For the expression of the antibody::pIII (phage protein III) fusion proteins *via* phage display, two different genetic approaches have been developed. Initially, the antibody genes were directly inserted into the M13 phage genome and fused upstream of the wildtype pIII gene (gIII) (McCafferty et al., 1990). Due to a better genetic stability and opportunity for independent regulation of phage and antibodies production, today the most successful system is based on phagemids. These are independent plasmids which provide the genes encoding the antibody::pIII fusion proteins and contain a phage morphogenetic signal for packaging of the phagemid into the phage particles (Breitling et al., 1991). In both phage display systems, the antibody fragments are always displayed on the phage surface and the corresponding antibody gene is packaged into the same phage particle. This successful technology was awarded with the Nobel Prize for Chemistry in 2018. For antibody phage display, the most common used antibody formats are the single chain fragment variable (scFv) (Vaughan et al., 1996; Schofield et al., 2007; Hust et al., 2011) or fragment antigen binding (Fabs) (de Haard et al., 1999; Hoet et al., 2005). Other used formats are human VH domains (dAbs), immunoglobulins of sharks (IgNARs) and the variable domains of camel heavy chains (VHHs) (Muyldermans, 2001; Nuttall et al., 2001; Holt et al., 2003; Nuttall et al., 2004; Muyldermans et al., 2009). The use of immune camel, llama or alpaca libraries gained popularity in the last five years. For veterinary research, chicken antibody libraries are often used (Fehrsen et al., 2005; Foord et al., 2007; Wemmer et al., 2010). The diversity of chicken antibody genes is the result of gene conversion. Here, the N- and C-terminal parts of chicken’s VH and VL are always identical, which facilitates antibody gene amplification and library cloning (Parvari et al., 1988; Chiliza et al., 2008).

**Figure 1:**
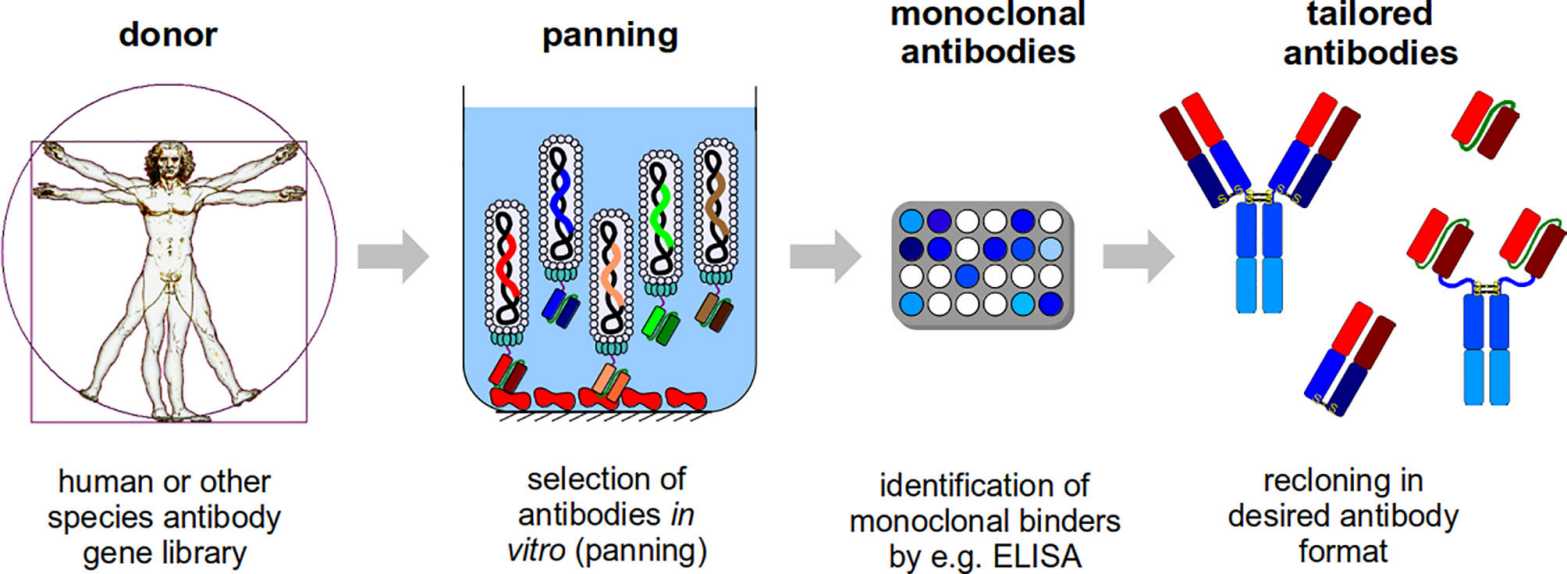
Schematic illustration of the antibody generation process. For the antibody generation, human phage display libraries or libraries from other species are used. These libraries will be used for the *in vitro* selection (panning) on a target molecule. In short: the antibody phage particles will be incubated with target molecule (in the illustration, a target molecule was immobilized in a microtitre plate well and a scFv phage display library is used), the non and weak binding antibody phage particles will be washed away and the binding antibody phage will be eluted (the procedure will be repeated 3-5 times) and further analyzed. In the next step, monoclonal binders will be identified, e.g. by ELISA. Finally, the selected antibody fragments can be recloned, because the corresponding gene is packaged in the phage particles, and produced in any antibody format tailored to the final application.

In the antibody selection process (panning), in most cases, the target molecules is immobilized on a solid surface, like plastic surfaces - normally used for ELISA - such as polystyrene tubes or microtiter plate wells, the latter of which is the most simple and commonly used method (Hust et al., 2002; Russo et al., 2018), affinity chromatography column matrixes (Breitling et al., 1991) or magnetic beads (Sawyer et al., 1997). An alternative is the use of biotinylated targets for a selection in solution followed by a “pull-down” with streptavidin beads (Schütte et al., 2009; Wenzel et al., 2020b). After incubating the entire diversity of the phage display library with these immobilized antigens, the tremendous excess of non- or weak-binding antibody phage particles are removed by stringent washing. Afterwards, the bound antibody phage will be eluted, mainly by trypsinization or by pH shift, and used for *E. coli* infection. Subsequently, the phagemid bearing *E. coli* will be infected with a helperphage to produce new antibody phage particles with an enriched fraction of antigen specific antibody phage clones. Usually 3-5 panning rounds are performed before screening. Specific binders can be identified in a screening process using monoclonal soluble antibodies or monoclonal antibody phage by e.g. ELISA (Frenzel et al., 2014), WB (Hust et al., 2002) or flow cytometer (Ayriss et al., 2007). The antibody fragment genes can be subcloned to produce antibodies in any desired antibody format, e.g. IgG or scFv-Fc (Hoet et al., 2005; Dübel et al., 2010; Hust et al., 2011; Jäger et al., 2013; Frenzel et al., 2014). **Figure 1** shows an overview on the phage display antibody selection process. An overview about phage display in comparison to other display technologies is given by Valldorf et al. (2021).

Depending the origin of the V-gene repertoires, there are two types of libraries: immune libraries and universal libraries. Immune libraries are constructed from convalescent patients or immunized donors. This library type is most used to obtain an antibody against a particular target, e.g. an infectious pathogen like Ebola virus (Maruyama et al., 1999a) or diphtheria toxin (Wenzel et al., 2020a). The advantage of this library type is that the V-genes contain hypermutations and are already affinity-matured for the targeted pathogen.

The alternatives are universal libraries - in the past also named “single-pot” libraries - made of naïve, semi-synthetic or synthetic gene repertoires. This library type allows to isolate antibody fragments binding to virtually every possible target without the need for an immunization or the availability of patients’ blood. (Winter et al., 1994; Dübel et al., 2010; Frenzel et al., 2017). In a pandemic situation, this provides a source for immediate antibody generation against a new arising pathogen, independently from the availability of material from patients (Bertoglio et al., 2021b). The Naïve libraries are cloned from rearranged V genes derived from B cells (IgM) of non-immunized donors. Examples for naïve libraries are the HAL scFv libraries (Hust et al., 2011; Kügler et al., 2015) and Fab library constructed by de Haard and colleagues (de Haard et al., 1999). Semi-synthetic libraries are cloned from unrearranged V genes from pre-B cells (germline cells) (Griffiths et al., 1994)⁠ or in most cases from one antibody framework (Pini et al., 1998)⁠. In this defined framework one or several CDRs, but always the CDR H3, are randomized, e.g. by using DNA-primers covering the CDR H3 section. The semi-synthetic libraries Tomlinson I and J libraries are often used and are the best example for semi-synthetic libraries. These libraries have a VH3-23 and Kappa IKV1-39 framework combined with randomized CDR2 and CDR3 (de Wildt et al., 2000). For the FAB310 antibody gene library, a combination of naïve and synthetic repertoire was used. Here, the Fd fragment (VH+CH1) contains randomized CDR1 and CDR2 in the human VH3-23 framework and a naïve CDR3 regions from autoimmune patients. The Fd fragments were combined with light chains also from autoimmune patients (Hoet et al., 2005). The transition of semi-synthetic to synthetic libraries is not strict. In general, complete synthetic libraries are made of human frameworks and here all CDR cassettes are synthetic, the best examples are the HUCAL libraries (Hayashi et al., 1994; Knappik et al., 2000; Tiller et al., 2013)⁠. Universal libraries have a theoretical diversity higher than 10^10^ independent clones (Schofield et al., 2007; Glanville et al., 2009; Lloyd et al., 2009; Tiller et al., 2013; Kügler et al., 2015).

*In vitro* display methods, like phage display, differ from classical animal immunization approaches to obtain polyclonals or hybridomas, in the possibility to directly generate sequence defined monoclonal antibodies. Additionally, the phage display antibody selection process occurs entirely *in vitro* and its customization allows to pre-filter antibodies with the intended binding properties, such as epitope specificity, binding at defined pH and temperatures, or absence of unwanted cross-reactivities. An alternative method that allows to generate sequence defined antibodies is the microfluidics-based B-cell selection method (Seah et al., 2018). B-cells secreted antibodies binding to labeled antigen material can be assessed at single cell level in droplets of buffer solution. This method, very laborious and technically demanding, is restricted to the availability of patient or immunized animal B-cells. Additionally, this approach provides a lower degree of flexibility in the number of assays which can be performed in the initial antibody screening phase. The upside of this method is the possibility to discover antibodies already in the IgG format. These features made this method particularly popular for the discovery of therapeutic antibodies from B-cells of disease-recovered patients. This is the case of the FDA approved Neutralizing antibodies to Ebola virus glycoprotein Ebanga (Ansuvimab-zykl) (https://www.fda.gov/drugs/drug-safety-and-availability/fda-approves-treatment-ebola-virus), the anti-HIV neutralizing antibodies under clinical evaluation (Spencer et al., 2021) or the anti-SARS-CoV-2 antibodies REGN10933 and REGN10987 (Hansen et al., 2020).

To date, 106 monoclonal antibodies and antibody conjugates have been approved by EMA and/or FDA (status March 2021), 87 antibodies are in clinical phase 2 or 3 and 122 antibodies are in phase 1 or IND filed (Source: antibodysociety.org, status April 2020). Most approved antibodies are for the treatment of autoimmune diseases and cancer. The annual sales of therapeutic antibodies exceeded 98 billion US$ in 2017 and the market will be predicted with 137-200 billion US$ in 2022 (Grilo and Mantalaris, 2019). The mode of action of therapeutic antibodies are numerous and include neutralization of substances e.g. cytokines like tumor necrosis factor alpha (TNF-α) (Alonso-Ruiz et al., 2008) or toxins (Rasetti-Escargueil et al., 2017), human cell binding and modulation of the host immune system (Chatenoud and Bluestone, 2007), blocking of receptors which are overexpressed on cancer cells like epidermal growth factor receptor (EGFR) (Peeters et al., 2009) or combinations of these mode of actions (Adams and Weiner, 2005). Currently, eight recombinant antibodies or antibody cocktails are approved for the direct treatment of pathogens or toxins. The first recombinant antibody approved for the treatment of an infectious disease was Palivizumab. It is a classical humanized antibody approved in 1998 for the treatment of Respiratory syncytial virus (RSV) bronchiolitis (Malley et al., 1998). For anthrax treatment, the human antibody Raxibacumab was developed. This antibody is derived from a human phage display library (made by Cambridge Antibody Technology in cooperation with Human Genome Science) and was approved in 2012 (Mazumdar, 2009; Kummerfeldt, 2014). A second antibody for post-exposure treatment of anthrax, the antibody Obiltoxaximab, has been approved in 2020 (Henning et al., 2018). A further approved antibody against toxins is Bezlotoxumab, approved in 2016. This antibody neutralizes the *Clostridioides difficile* toxin TcdB (Babcock et al., 2006; Johnson and Gerding, 2019). The anti-HIV antibody Ibalizumab has been approved in 2018. The mode of action is very unique for an anti-viral antibody, because it blocks cell entry by binding to the human receptor CD4 on the target cells instead of binding to the virus (Beccari et al., 2019). The anti-rabies antibody Rabishield to replace anti-rabies serum therapies has been approved 2018 in India (Wang et al., 2011; Gogtay et al., 2018). End of 2020, two products were approved for the treatment of Ebola: the three-antibody cocktail REGN-EB3 (Atoltivimab/maftivimab/odesivimab) (Pascal et al., 2018) and the single antibody mAb114 (Ansuvimab-zykl) (Corti et al., 2016). These antibodies showed a good efficiency in clinical trials against Ebola virus especially in comparison to Remdesivir (Mulangu et al., 2019). In the COVID-19 pandemic, the anti-SARS-CoV-2 antibody cocktail Casirivimab (REGN10933)/Imdevimab (REGN10987) (Baum et al., 2020; Hansen et al., 2020) and the antibody Bamlanivimab (LY-CoV555) (Chen et al., 2021) got a conditional marketing authorization in Europe and an Emergency use authorization (EUA) in the USA for non-hospitalized patients with mild to moderate symptoms. The FDA also authorized the antibody cocktail Bamlanivimab and Etesevimab (JS016, CB6) (Shi et al., 2020) in the USA for the same indication. For the treatment of hospitalized patients with moderate to severe symptoms, phage display derived antibody COR-101 (STE90-C11) against SARS-CoV-2 (Bertoglio et al., 2021a) is in clinical phase 1b/II.

In the next sections, we provide an overview of recombinant antibodies derived from phage display against bacterial and viral pathogens, eukaryotic pathogens (parasites, fungi) and toxins, as well as detailed examples for diagnostics and therapy.

## Recombinant Antibodies Against Bacteria

Toxins are the most frequent antigenic target of therapeutic antibodies used to fight bacterial pathogens. This topic is thoroughly discussed in a dedicated paragraph: “targeting of toxins with recombinant antibodies”. Excluding toxin antigens, antibodies raised against bacteria are predominantly used in diagnostics (Skottrup et al., 2011; Ferrara et al., 2012) and to assess the presence of contamination in environmental samples (Griep et al., 1998; Mechaly et al., 2008). As of today, microbiologic bacteria identification by cultivation remains the benchmark approach for the diagnostic of several pathogenic bacterial species, including *Mycobacterium tuberculosis* (Norbis et al., 2014), *Salmonella* Typhimurium (Kuhn et al., 2012), or *Listeria monocytogenes* (Gasanov et al., 2005).

The many drawbacks of these methods are the low throughput and high costs caused by their inherently long experimental duration, the higher risk of exposure to pathogenic organisms and the need of highly trained personnel. Real-time PCR and other molecular diagnostics approaches have been introduced to reduce the time needed for the identification of many bacteria (Liang et al., 2018), while offering high detection sensitivity and specificity. Nevertheless, such methods most often depend on the availability of laboratory infrastructure and, if portable, are less amenable to high throughput parallelized measurements (Xu et al., 2020). Similarly, mass spectrometry is able to provide high sensitivity, however, this technology requires expensive devices and trained personnel (Dixon et al., 2015). On the other hand, antibody-based detection methods such as ELISA and immunochromatography, are faster, simpler, and well-suited for high-throughput. These features, together with the lower costs compared to other techniques, contributed to the success of these diagnostic approaches world-wide, including developing countries, where resources may be scarce and laboratories sparsely distributed over the territory.

Up to date, a vast plethora of antibodies for diagnostic applications has been generated *via* phage-display against different bacterial proteins or carbohydrates (**Table 1**). Interestingly, research on this field seems to be in constant rise, as 44% of these antibodies have been generated only in the past five years.

**Table 1:** Recombinant antibodies derived by phage display against pathogenic bacteria.

Bacteria	Target	Library Type	Antibody Format	Antibody Origin	Application	Reference
*Acinetobacter baumannii*	Bap	Immune	VHH	camel	ELISA, WB, *in vivo* neutralization	(Payandeh et al., 2014)
*Bacillus anthracis*	S-layer protein EA1	immune	VHH	llama	ELISA, WB	(Walper et al., 2012; Anderson et al., 2018)
*Bacillus anthracis*	live bacteria	immune	scFv	mice	ELISA, WB, IF	(Mechaly et al., 2008)
*Bacillus anthracis*	protective antigen (PA)	naïve	scFv	human	ELISA, Inhibition assay	(Ahn et al., 2019)
*Bordetella pertussis*	filamentous hemagglutinin,pertactin	immune	scFv	mice	ELISA, *in vitro* inhibition, *in vivo* studies	(Hussein et al., 2007)
*Brucella melitensis*	radiated bacteria	immune	scFv	mice	ELISA	(Hayhurst et al., 2003)
*Campylobacter jejuni*	whole cells	immune	scFv	rabbit	ELISA, WB	(Nzuma et al., 2018)
*Chlamydophila psittaci*	2.4[2.8]2.4-linked Kdo tetrasaccharide	immune	scFv	mice	ELISA, IF	(Gerstenbruch et al., 2010)
*Chlamydia trachomatis*	elementary bodies	naïve	scFv	human	ELISA, WB, IF	(Sheets et al., 1998; Lindquist et al., 2002)
*Chlamydia trachomatis*	unknown	immune	VHH	camel	unknown	(Tillib et al., 2017)
*Clostridium botulinum*	RhoA/B	naïve	scFv	human	WB, immunofluorescence	(Rohrbeck et al., 2016)
*Clostridium difficile*	different surface proteins (including FliC and FliD)	semi-synthetic	scFv	human	ELISA, WB, *in vitro* motility assay	(Shirvan and Aitken, 2016)
*Clostridium difficile*	Surface layer proteins (SLPs)	immune	VHH	Llama	ELISA, WB, *in vitro* motility assay	(Kandalaft et al., 2015)
*Escherichia coli*	live *E. coli* F17 +	immune	VHH	camel	ELISA	(Salhi et al., 2020)
*Francisella tularensis*	lipopolysaccharide (LPS)	immune	scFv, IgG	rabbit	ELISA, WB, IF, *in vitro* neutralization	(Mechaly et al., 2019)
*Klebsiella pneumoniae*	different strains of K. pneumonia (possibly MrkA)	naive	scFv, scFv-Fc, IgG	human	ELISA, OPK, IF, *in vivo* protection	(Wang Q. et al., 2016)
*Haemophilus influenzae*	capsular polysaccharide	immune	Fab	human	ELISA	(Reason et al., 1997)
*Helicobacter pylori*	urease	semi-synthetic	scFv	human	ELISA, WB, WB	(Fouladi et al., 2019)
*Helicobacter pylori*	bacterial lysate	immune	scFv	human	ELISA	(Reiche et al., 2002)
*Lactobacillus acidophilus*	S-layer protein	naïve	scFv	human	FACS, WB	(Close et al., 2013)
*Lawsonia intracellularis*	live bacteria	semi-synthetic	scFv	human	ELISA, IF	(Dezorzová-Tomanová et al., 2007)
*Legionella pneumophila*	whole cells	naïve	scFv	human	ELISA, biosensor	(Kuhn et al., 2017)
*Leptospira spec.*	LipL21	naïve	scFv	human	ELISA	(Mohd Ali et al., 2021)
*Listeria monocytogenes*	internalin B	immune/naïve	VHH	alpaca, llama, camel	ELISA	(Gene et al., 2015)
*Listeria monocytogenes*	pyruvate dehydrogenase complex-enzyme 2 (PDC-E2)	naïve	scFv, scFv-Fc	human	ELISA, WB, IF	(Moreira et al., 2020)
*Listeria monocytogenes*	heat-killed cells	naive	VHH	Llama	ELISA, WB	(Tu et al., 2016)
*Moraxella catarrhalis*	HMW-OMP	semi-synthetic	scFv	human	ELISA, FACS, WB, *in vitro* inhibition	(Boel et al., 1998)
*Mycobacterium avium*	cell lysate	immune	scFv	sheep	ELISA, WB, FACS, IF	(Berger et al., 2006)
*Mycobaterium bovis*	HSP65	semi-synthetic	scFv,scFv-IgY-Fc	chicken	ELISA, WB	(Wemmer et al., 2010)
*Mycobacterium leprea*	PGLI-M3	naïve?	scFv	human	ELISA, immunohistochemistry	(Lima et al., 2020)
*Mycobacterium tuberculosis*	antigen 16kDa (HspX)	semi-synthetic	scFv	chicken	ELISA	(Sixholo et al., 2011)
*Mycobacterium tuberculosis*	antigen 85B	naïve	scFv,scFv-Fc	human	ELISA, WB, lateral flow strip assay	(Fuchs et al., 2014)
*Mycobacterium tuberculosis*	antigen 85	naïve	scFv,scFv-Fc	human	ELISA	(Ferrara et al., 2012)
*Mycobacterium tuberculosis*	LAM	immune	scFv	Rabbit/chicken	Sandwich-ELISA	(Kawasaki et al., 2019)
*Mycobacterium tuberculosis*	α-crystalline antigen	immune	scFv	human	ELISA	(Hamidon et al., 2018)
*Mycobacterium tuberculosis*	LAM	immune	scFvIgG	rabbit	ELISA, WB, dot blot, flow cytometry	(Yan et al., 2020)
*Neisseria meningitidis*	NadA	Immune	VHH	llama	Dot blot, WB, IHC, blocking assay, *in vitro* model	(Kulkarni et al., 2020)
*Porphyromonas gingivalis*	RgpB	naïve	VHH	camel	ELISA	(Skottrup et al., 2011)
*Pseudomonas aeruginosa*	SpuE	naïve	scFv	human	ELISA	(Zhang Y. et al., 2019)
*Pseudomonas aeruginosa*	flagelar capping protein (FliD)	naïve	scFv, scFv-Fc	human	WB	(Postel et al., 2016)
*Ralstonia solanacearum*	LPS	naïve	scFv	human	ELISA, IF, WB	(Griep et al., 1998)
*Salmonella* Typhimurium	OmpD	naïve	scFv	human	ELISA	(Meyer et al., 2011)
*Salmonella* Typhimurium	5 different immunogenic proteins	naïve	scFv	human	ELISA	(Meyer et al., 2012)
*Staphylococcus aureus*	FnBPA, ClfA	immune	scFv	bovine	ELISA, WB, inhibition assay	(Wang M. et al., 2015, Wang M. et al., 2016)
*Staphylococcus aureus*	cells	immune	scFvscFv-Fc	human	ELISA, dot blot	(Nian et al., 2016)
*Streptococcus pneumoniae*	Pep27	synthetic	scFv	human	ELISA	(Kim et al., 2018)
*Streptococcuspneumoniae*	PspA	synthetic	scFv	human	WB	(Jang et al., 2017)
*Streptococcus mutans & sobrinus*	live cells	synthetic	Fab	human	ELISA, IF, Flow cytometry*, in vitro* biofilm assay, *in vivo* model	(Alam et al., 2018)
*Vibrio cholera*	LPS	immune	VHH	camel	ELISA, *in vivo* challenge	(Ebrahimizadeh et al., 2013)
*Vibrio parahaemolyticus*	VP1694	immune	scFv	mice	ELISA, WB	(Wang R. et al., 2014)
*Vibrio parahaemolyticus*	OmpU	semi-synthetic	sdAb	human	ELISA, *in vitro* inhibition	(Yu et al., 2020)
*Yersinia pestis*	F1	naïve	scFv	human	ELISA	(Lillo et al., 2011)

ELISA, enzyme linked immunosorbent assays; IHC, immunohistochemistry; IF, immuno fluorescence; FACS, fluorescence-activated cell sorting; WB, western blot.

In the following paragraphs, we offer detailed examples on antibodies against different bacterial pathogens that were generated using phage display.

*Mycobacterium tuberculosis* (Mtb) is the bacteria responsible for a lung infection known as Tuberculosis (TB). This pathogen specifically attacks and replicates inside the macrophage cells in the alveoli, causing fatigue, fever and coughing (Fu and Fu-Liu, 2002). As of 2021, this disease affected approximately 10 million people and caused 1.5 million deaths worldwide per year (World Health Organization, 2021). TB early diagnosis is essential to improve treatment and limit disease spread. The protein secreted by Mtb in the highest amounts, Antigen 85 (Ag85), represents an ideal target for diagnostics (Wiker and Harboe, 1992). Ag85 is an oligomeric protein composed of three relatively small (30-32 kDa) homologue proteins (Ag85A, Ag85B and Ag85C) (Wiker et al., 1992). In a novel approach described by Ferrara et al. (2012), a combination of antibody display techniques was successfully adopted to select Ag85 complex-specific antibodies. A universal phage display naïve scFv-library with a nominal diversity of ~10^11^ unique antibodies was used for initial selection on Ag85, obtaining a sub-library of 10^5^ pre-selected antibodies. Second, the selected antigen-specific scFv genes were used for the generation of yeast-display antibody sub-library. Upon completion of yeast based antibody generation, each clone was tested in FACS for its ability to specifically recognize the antigen target resulting in the discovery of 192 clones having the desired properties. Sequence analysis revealed that 111 of these antibodies were unique and could be used in further Ab characterization. Chessboard antibody combination testing resulted in the identification of 7 antibody pairs that detect as little as 22.7 nM Ag85 in patient sera or 6.1 nM in serum free-conditions. The uniqueness of this work relies in the fact that each validated antibody was specific for the Ag85-complex only. Other approaches were aimed at discovering subunit-specific antibodies, as in the case of Fuchs et al. Here, the naïve HAL7/8 phage display libraries were incubated with recombinant Ag85B subunit (Fuchs et al., 2014). The resulting antibodies were shown to have, in sandwich ELISA or lateral flow test, an antigen protein limit of detection of respectively 10 ng/mL and <5 ng/mL. Nevertheless, when using bacterial cell extracts or filtered culture material, immunodetection could be only performed in ELISA or western blot. The authors concluded that the absence of an antibody affinity improvement step may have been the reason for their results. This supposition found indirect confirmation in a different study by Sixholo and colleagues (Sixholo et al., 2011) describing the selection of antibodies against the Mtb 16 kDa heat shock protein X (HspX) from chicken semi-synthetic Nkuku antibody gene library. By the repetition of four panning rounds against recombinant antigen material, three unique scFv’s were confirmed to bind HspX in ELISA. On purpose, the clone with the weakest binding was chosen for *in vitro* affinity maturation by different methods. In the first approach, a mutant library of the parental antibody gene was generated by error-prone PCR, resulting in a diversity of ~ 3 × 10^7^ unique scFv clones. Repeating the antibody selection using this library under more stringent conditions, led to the discovery of three mutant scFv’s with increased binding affinity. Direct binding comparison in ELISA showed that the newly generated antibodies produced a signal ~11 times higher than the parental clone. The result was confirmed by the improved association and dissociation kinetics in SPR analysis. All affinity-improved antibody genes contained mutated amino acids in both CDR and framework regions. In the second approach, a glycine residue alone was replaced to the common 15-mer linker connecting the VH to VL, resulting in tetramer formation (Schmiedl et al., 2006). The avidity effect leading to cooperative binding resulted in a binding signal increase of ~14 times in ELISA. Antibodies against Mtb were also generated by Hamidon and colleagues (Hamidon et al., 2018) utilizing a different approach. A patient immune TB library with a nominal diversity of 10^9^ different scFv antibodies could be generated starting from the B-cell material from six individuals infected with TB. This immune library was used to select antibodies against Mtb α-crystalline, resulting in three unique antigen-specific clones validated for antigen binding in ELISA and Western Blot assays.

Periodontitis is a disease where inflammation of the dental gum results in its progressive retraction and, if not cured on time, loss of teeth. One of the major pathogens involved in gum infection is *Porphyromonas gingivalis* (Socransky et al., 1998). The RgpB cysteine proteases secreted by this pathogen is one of the causing factors of the gum inflammation and constitutes a valid biomarker candidate (Imamura, 2003). Skottrup et al. (2011) generated antibodies against immobilized RgpB from a naïve library of camelid antibodies with a diversity of ~5 x 10^7^ different clones. Such antibodies consist of a monomeric VHH domain (~15 kDa), which offer multiple advantages for diagnostics, in particular high yields in *E. coli* production systems, good stability and small epitope accessibility. This library yielded an elite antibody clone with 362 pM affinity towards the recombinant RgpB, also specific to the antigen presented on cells. While not inhibiting RgpB catalytic activity, this antibody showed in subtractive inhibition ELISA a limit of detection equal to ~8 x 10^6^ cells/mL of saliva.

In the field of foodborne gastrointestinal infection causing pathogens, *Salmonella* Typhimurium is probably the most studied bacteria (Jacobsen et al., 2011). In the attempt to generate antibodies for the detection of this pathogen, Meyer et al. (2012) utilized ORFeome phage display for biomarker discovery. Starting from bacterial genomic material, a random protein fragments library was screened using sera from *Salmonella* infected animals. In a second step, the antigens corresponding to the newly discovered immunogenic proteins were used to select human antibodies from the naïve HAL gene libraries. Antibodies are also potent tools for the monitoring of a vaccination campaign and discrimination of infected from vaccinated subjects. For the development of DIVA (Differentiating Infected from Vaccinated Animals) vaccines against *S. *Typhimurium, Selke and colleagues (Selke et al., 2007) generated an engineered bacterial strain lacking the marker protein OmpD (outer membrane protein D). A serological test using OmpD-specific antibodies constitutes a tool to discriminate infected animal from vaccinated ones. Meyer et al. (2011) generated scFv antibodies against OmpD for antigen detection in competitive ELISA on swine serum.

Another very prominent foodborne pathogen is *Listeria monocytogenes*. This bacterial infection, listeriosis, can cause severe illness, including meningitis and sepsis when reaching the systemic circulation. As of today, 13 different *L. monocytogenes* serotypes have been discovered, of which type 4b, 1/2a and 1/2b have shown to be the most virulent (Datta and Burall, 2018). Contaminated food also contains non-pathogenic *Listeria* species, making the diagnosis more difficult. Indeed, their faster growth rate during the enrichment step required for bacteria detection is the major cause of false-negative results in *Listeria monocytogenes* food diagnostics (Besse et al., 2010). Hence, species-specific immunodiagnostic tools constitute a necessary resource to improve both *L. monocytogenes* as well as *Listeria* spp. detection. Gene and colleagues (Gene et al., 2015) used naive and immune camelid antibody libraries for the selection *via* phage display of diagnostic nanobodies against internalin B (InIB), a protein which is strongly involved in host-cell invasion. Two of the five generated nanobodies, R3-03 and R3-30, showed picomolar binding affinities to InIB, representing a valuable resource for bacteria immunodetection in poorly concentrated samples. A recent study from Moreira et al. (2020) described the use in combination of different phage display techniques to identify new *Listeria* spp. protein biomarkers and rise antibodies against them. Initially, the human naïve antibody display phage library HAL9/10 was used to generate antibodies against different cell fractions from *L. monocytogenes* (cell wall, membrane, and cytoplasm). This way, four binders were isolated, which allowed the identification of a novel target for *Listeria* spp. detection named pyruvate dehydrogenase complex-enzyme 2 (PDC-E2) by using both immunomagnetic separation/mass spectrometry (IMS-MS) and ORFeome phage display. One of these antibodies was also used in immunofluorescence on non-permeabilized cells confirming the surface localization of the target. Antibody selection against PDC-E2 recombinant antigen yielded 16 additional antibodies. These 20 antibodies were finally tested in indirect ELISA against a panel of 17 *Listeria* species (including the most virulent *L. monocytogenes* serotypes 4b, 1/2a, and 1/2b) and 16 non-*Listeria* species. Two antibodies, GSM313-E9 and GSM313-H8, provided 100% sensitivity and specificity for *Listeria* spp. detection. Moreover, the binding region (epitope) of 18 out of the 20 antibodies was identified *via* single-gene phage display for epitope mapping, enabling to define the lipoyl domains of PDC-E2 as the major contributors for the detection through this target.

## Recombinant Antibodies Against Viruses

Viruses are in every kind of environment and are able to infect any sort of host. New viral species are continuously discovered and therefore much research has been devoted during the recent decades to generate vast sets of antibodies against viruses, both from universal and immune libraries using phage display technology. Target for selection of antibodies ranged from peptides and recombinant viral proteins to complete viral or pseudoviral particles, leading to the isolation of antibodies against human pathogenic viruses such as Sin nombre virus (Velappan et al., 2007), Dengue virus (Cabezas et al., 2008; Saokaew et al., 2014), Influenza virus (Lim et al., 2008; Sui et al., 2009), VEEV (Kirsch et al., 2008), Norovirus (Higo-Moriguchi et al., 2014), SARS coronavirus (Sui et al., 2004), SARS-CoV-2 (Bertoglio et al., 2021b) or Hepatitis C (Songsivilai and Dharakul, 1998) from naïve antibody gene libraries. Alternatively, another source of monoclonal antibodies has been immune antibody gene libraries constructed from hosts infected with e.g. Hantavirus (Liang et al., 2001; Koch et al., 2003), WEEV (Hülseweh et al., 2014), HIV (Mohammadzadeh et al., 2014; Trott et al., 2014), SARS (Kang et al., 2006), Yellow fever virus (Daffis et al., 2005) or Influenza virus (Throsby et al., 2008; Sun et al., 2009). Additionally, semi-synthetic libraries have been used to generate antibodies against e.g. Influenza virus (Avnir et al., 2014). Plant viruses have also been addressed for antibody selections (Ziegler et al., 1995; Hust et al., 2002; Orecchia et al., 2008), beside human and animal viruses.

Virus-specific antibodies have also been selected from libraries originating from different species such as those from macaque (Rülker et al., 2012), chimpanzee (Goncalvez et al., 2008), llama (Fatima et al., 2014), mouse (Liu H. et al., 2014), chicken (van Wyngaardt et al., 2004) and human origin (Tikunova et al., 2012).

The predominant format harnessed for initial selection of antibodies against viruses has been scFv format (Gould et al., 2005; Wu et al., 2014), although Fab (Wyrzucki et al., 2014; Zhao et al., 2014) and VHH libraries (Fatima et al., 2014) were often used. In a peculiar case, Xiao et al. used the antibody CH2 domain as scaffold to generate binders against gp120 of HIV (Xiao et al., 2009). In the case of HIV, some unique aspects could be highlighted as for example that the neutralizing anti-gp41 antibody HK20 has a higher neutralization potency as scFv or Fab compared to IgG, indicating that the IgG format has more difficult access to the epitope, due to the formats bigger steric hindrance (Sabin et al., 2010). Another noteworthy example is the anti-gp41 VHH 2H10, where a CDR3 tryptophan is not involved in epitope binding, but resulted essential for virus neutralization (Lutje Hulsik et al., 2013).

In the following paragraphs, we present detailed examples for antibody generation and engineering using phage display against different viral *taxa*.

Vaccinia virus is the arketypal virus within the genus of O*rthopoxvirus*. This virus is with 300x 230 nM a large DNA virus with a genome of about 0,2 Mb (Malkin et al., 2003). Among the species included in O*rthopoxvirus* genus, there are monkeypox virus, cowpox virus and especially variola virus, the causative agent of smallpox in humans. Occurrence of natural smallpox has been eradicated in 1977 thanks to a massive WHO vaccination program, which started ten years earlier. However, since no vaccination of the general population is nowadays conducted and the use of variola virus as potential threat following intentional release has renewed the research interest for safe and effective smallpox vaccines, due to case fatality rates of 30% or more among unvaccinated subjects (Henderson et al., 1999). It is assumed that immunity against one poxvirus provides broad immunity against most of the viral family members given the high similarity shared among orthiopoxviruses (Earl et al., 2004; Berhanu et al., 2008). Using an immune scFv phage display library constructed from vaccinia virus immunized patients, human vaccinia specific antibodies were isolated. *In vitro* plaque-reduction neutralization tests proved that seven of these antibodies were able to neutralize both, vaccinia and cowpox virus. Five of them, additionally neutralized monkeypox virus (Tikunova et al., 2012). Another set of antibodies were selected from a Fab immune library derived from chimpanzee, vaccinated with vaccinia virus. After conversion into a chimeric chimpanzee/human IgG format, antibody 8AH8AL, binding to vaccinia protein B5, was neutralizing *in vitro* for vaccinia and smallpox virus. Its protective effect was assessed in mice challenged with vaccinia virus even when the monoclonal antibody was administered 2 days after challenge. In this model, 8AH8AL provided significantly greater protection than that of the previously isolated rat anti-B5 antibody 19C2 (Chen et al., 2006). Since experimental work with smallpox virus is extremely restricted and essentially not allowed, vaccinia had to be used as model.

Ebola Virus and Marburg Virus, two members of the *Filoviridae* family, cause severe hemorrhagic fevers and extremely high mortality of up to 90% in infected humans. Besides the obvious public health concern associated with its natural outbreaks, Ebola virus might be considered a potential agent of biological warfare and bio-terrorism (Goodchild et al., 2011). Phage display-derived human antibodies targeting Ebola virus were selected from an immune library originated from survivors of the 1995 Ebola virus outbreak in Kikwit, Democratic Republic of Congo (Maruyama et al., 1999b). Nucleoprotein, envelope glycoprotein and secreted envelope glycoprotein binders have been isolated in this study. Antibody (KZ52), recognizing the envelope glycoprotein, neutralized *in vitro* both, as Fab (50% neutralization at 0.4 µg/mL) as well as full IgG (90% neutralization at 2.6 µg/mL) (Maruyama et al., 1999a). Follow-up studies demonstrated effective protection *in vivo* in a guinea pig Ebola challenge model, upon administration of the antibody up to one hour post viral challenge (Parren et al., 2002). Unfortunately, KZ52 showed no protection in macaques challenged with Ebola, even though the antibody was given in a two-dose regimen with the first dose one day prior viral challenge and the second after three days the former dose (Oswald et al., 2007). Further examples of antibodies selected through phage display against *Filoviridae* family members encompass a murine scFv and two shark IgNAR V immune libraries generated against inactivated Zaire Ebola virus to yield several antibodies specific for the viral matrix protein VP40 and the viral nucleoprotein (Goodchild et al., 2011). Of note, this work is the first example of a successful targeted IgNAR V isolation from a shark immune library. The antibody combination X10B1/X10H2 generated against the glycoprotein of Ebola Sudan virus (SUDV) showed 100% *in vivo* protection in scFv-Fc format in a mice challenge model. After 35 days post infection, the mice were challenged again with SUDV and all mice survived showing the development of an own protective immune answer against SUDV (Froude et al., 2017). These antibodies were also tested as IgG and the X10H2 antibody was successfully employed in a cocktail with the non-phage display derived antibody 16F6 (Herbert et al., 2020). Recombinant antibodies against Marburg virus were generated using human synthetic libraries (Amatya et al., 2019) or macaque immune libraries (Froude et al., 2017). The macaque antibodies showed *in vitro* neutralization and up to 100% *in vivo* protection against wt Ci67 Marburg virus in a prophylactic mice challenge model using 100 µg scFv-Fc/mice.

*Flaviviridae* family member Dengue virus (DENV) causes at least 100 million symptomatic infections each year and became a major health and economic burden in over 50 countries worldwide (Moreland et al., 2010; de Alwis et al., 2012; Moreland et al., 2012). Its genome encompasses a single open reading frame, contained in the ~ 11 kb positive strand RNA. The four circulating serotypes of DENV show approximately 70% sequence homology (Moreland et al., 2012; Zhao et al., 2014). DENV infection can result in clinical symptoms that include dengue fever, dengue hemorrhagic fever (DHF) and dengue shock syndrome (DSS) (Yamanaka et al., 2021), which is presumably promoted by antibody-dependent enhancement (ADE) (Taylor et al., 2015). ADE results from partially or non-neutralizing anti-dengue IgG antibodies from a former infection mainly of another dengue serotype. The presence of ADE is associated with the onset of DHF that can be fatal (Halstead, 2014). Two Fabs, namely 5H2 and 5D9, were isolated from a chimpanzee immune library and neutralized dengue type 4 virus in a plaque reduction neutralization test (Men et al., 2004). In another example, human scFv antibodies specific to dengue virus envelope protein were selected by panning against recombinant full length envelope protein and its domain III (Saokaew et al., 2014). Because DENV envelope protein plays an essential role in virion assembly and viral entry, scFvs selected in this study were shown to block DENV infection *in vitro* (Saokaew et al., 2014). Dengue nonstructural protein 5 (NS5) is involved in viral replication and host immune response modulation. A naïve human Fab-phage library was screened for NS5-specific antibodies using various NS5 variants from Dengue Virus serotypes 1-4 as antigens for panning and characterization (Zhao et al., 2014). Alternating the use of NS5 derived from different dengue serotypes for each selection round, this strategy allowed the identification of two monoclonals that are cross-reactive against all four dengue serotypes. Another study presented antibodies selected against antigen Dengue virus particles directly captured from supernatant of infected Vero cells. Here, highly serotype specific antibodies were generated. Seven out of nine antibodies were specific to only one DENV serotype. One Dengue-3 selected clone cross-reacted with Dengue 1, whereas another clone showed pancross-reactivity despite being selected on Dengue 2 particles. Interestingly, all of the obtained antibodies recognized several strains of distinct genotypes within the corresponding serotype (Cabezas et al., 2008). Antibody C9 was isolated after panning against dengue envelope protein using a mouse/human chimeric Fab library. The antibody crossreacts with DENV1-3 and neutralizes DENV2 in cell-based assays after conversion into full length IgG (Moreland et al., 2012). Besides scFv and Fab, also variable domain heavy-chain antibodies (VHH antibodies) have been selected using phage display technology to target DENV2 NS1 protein. After four rounds of panning, 20 hits were selected (Fatima et al., 2014). A VHH antibody combination was developed which allows the detection of NS1 of all four Dengue strains (Shriver-Lake et al., 2018). For the therapy of dengue, the application of full-length antibodies like IgG may not provide the best solution for dengue therapy in the context of ADE. Antibodies with a silent Fc part or antibody fragments would be the preferred option for the development of therapeutic antibodies against dengue.

Venezuelan equine encephalitis virus (VEEV), an alphavirus of the *Togoviridae* family, causes both equine epidemics and also encephalitis in humans (Johnson and Martin, 1974; Weaver et al., 2004). Much research addressed the generation of neutralizing antibodies against VEEV since it is classified as Category B agent by the Centers for Disease Control and Prevention (CDC). An immune library from human donors was used as source for selection of mAbs targeting both VEEV envelope glycoproteins E1 and E2 (Hunt et al., 2010). The isolated Fabs L1A7 and F5 were neutralizing *in vitro*, with F5 being 300 times more effective than L1A7. Subsequently, F5 in full IgG format was employed to generate neutralization-escape variants of VEEV for epitope mapping purposes. Another study harnessed an immune macaque library to generate human-like antibodies. Among these antibodies, scFv-Fc ToR67-3B4 was protective in mice when administered 6 h post viral challenge with VEEV Trinidiad strain, allowing 80 to 100% survival after a challenge with 100-fold LD_50_. Counterintuitively, scFv-Fc ToR67-3B4 was able to neutralize other VEEV strains *in vitro*, but not Trinidad one. This tests proved that *in vitro* neutralization is not mandatory for an antibody to be actually protective in more complex infection models (Rülker et al., 2012). A further study described the antibody selection from a human naïve scFv gene library using complete, active VEEV particles as target. Specific detection of the VEEV strains TC83, H12/93, and 230 by the isolated antibodies was verified. Remarkably, none of the selected scFv phage clones showed cross-reactivity with Alphavirus species of the Eastern equine encephalitis virus (EEEV) and Western equine encephalitis virus (WEEV) antigenic complex or with Chikungunya virus (CHIKV), making them ideal tools for immunological detection and diagnostic of Alphavirus species (Kirsch et al., 2008). From WEEV immunized macaques, two different scFv antibody gene libraries were constructed. Reformatted as scFv-Fc, three antibodies originating from these libraries specifically bound WEEV in ELISA with little or absent cross-reactivity with other alphaviruses. Additionally, their neutralization potency was assessed *in vitro*. This was indeed the first study in which *in vitro* neutralizing antibodies against WEEV were developed. About 1 ng/mL of the best antibody (ToR69-3A2) neutralized 50% of 5 x 10^4^ TCID50/mL WEEV (Hülseweh et al., 2014). Four anti-WEEV antibodies from this study were also tested in an *in vivo* WEEV aerosol challenge approach. A dose dependent protection was measured. Interestingly, not only the best neutralizing antibody ToR69-3A2 was protective *in vivo*, but also the non-neutralizing antibody ToR68-2C3. The mode of action of the non-neutralizing antibodies cannot be the blocking of the virus host cell receptor interaction, but protection may results from complement-mediated lysis, antibody-dependent cell-mediated lysis of infected cells or opsonization followed by uptake by phagocytes (Burke et al., 2018). Single domain antibodies generated against E3E2 of WEEV from a llama immune library allowed the development of a MagPlex sandwich immunoassay to detect specifically WEEV and discriminate from other alphaviruses (Liu et al., 2018).

Influenza viruses are the etiologic agents of seasonal flu, a worldwide spread disease. In recent years, the “bird flu” (H5N1) and the pandemic “swine flu” (a variant of H1N1) moved into the spotlight of public attention and also of research focus, given the global spread of these variants. Due to many genetic events, such as e.g. antigenic drift and shift, new influenza variants will keep occurring in the future and will challenge vaccine and diagnostic development (Webster and Govorkova, 2014; Trombetta et al., 2015; Keilman, 2019). Sui et al. (2009) selected antibodies from a naïve scFv library against the H5 hemagglutinin ectodomain. Hemagglutinin is a trimer and the extracelluar part consist of a stalk domain and a globular head domain (Krammer and Palese, 2013). They identified ten antibodies binding to the trimeric H5, nine of which shared the same germline framework (VH1-69). These antibodies were converted into IgG1 and were protective in mice at dose of 10 or 15 mg/kg in both a prophylactic and therapeutic challenge models, respectively. Very remarkably, some antibodies cross-neutralized H1, H2, H5, H6, H8, and H9 influenza strains. These phage display derived antibodies are good candidates for broad-spectrum influenza immunotherapy (Sui et al., 2009).

Rabies is caused by the rabies virus which infects the central nervous system, always resulting in fatal disease, before Louis Pasteur developed the rabies vaccination. The current post-exposure therapy is based both on vaccination and polyclonal anti-rabies sera (Goudsmit et al., 2006; Briggs, 2012; Crowcroft and Thampi, 2015). From two different immune scFv libraries, 147 unique recombinant antibodies were selected against the rabies glycoprotein (Kramer et al., 2005). The neutralization of the selected mAbs was tested *in vitro* using 27 different street rabies virus isolates and the best neutralizing antibodies were further investigated *in vivo* in a hamster rabies (Bakker et al., 2005). This antibody was further analyzed with another human antibody, termed CR57, derived by somatic cell hybridization technique, in combination (Dietzschold et al., 1990), both in *in vitro* and *in vivo* models (Goudsmit et al., 2006). In a clinical phase 1 study, the safety and efficacy of the mAb cocktail (named CL184) was tested (Bakker et al., 2008) and subsequently in phase 2 studies. The antibodies were renamed Rafivirumab (CR57) and Foravirumab (CR4098). The *in vivo* efficacy in a hamster model was demonstrated with several rabies isolates (Franka et al., 2017). The antibody Rabishield, which is not derived by phage display, has been approved in 2018 (Wang et al., 2011; Gogtay et al., 2018).

The emergence of new viral species able to leak from their animal reservoirs and lastly infect the human host has been described several times in the last decades and has led to either local epidemics or global pandemics. In this regard, *Henipavirus* and *Betacoronavirus* genera, respectively, are emblematic examples of zoonotic viral diseases that posed serious threats to human health. The generation of monoclonal antibodies through phage display targeting these pathogens has been mainly investigated towards the development of new therapeutic agents, even though in the case of SARS-CoV-2 monoclonal antibodies are of foremost interest also for diagnostic purposes.

Henipaviruses known to be pathogenic to the human host are Hendra virus (HeV) and Nipah virus (NiV), that caused small outbreaks in Australia and Malaysia, respectively, first recognized during 1990 (Weatherman et al., 2018). Bats are the animal reservoir of these viruses and their transmission to human beings is either mediated through amplifying hosts, like pigs or horses, or direct, as documented in NiV Bangladesh 2001 outbreak, through contact between same contaminated food (Halpin et al., 2011; Weatherman et al., 2018). The infection of these negative-stranded RNA viruses causes mainly severe respiratory and neurological symptoms and sequelae with high mortality rate (Wong and Tan, 2012). The initial contact of these virus to host cells is mediated by attachment to glycoprotein G (also known as RBP -Receptor Binding Protein-), that binds the cellular surface proteins ephrinB2 or ephrinB3 (Bonaparte et al., 2005; Negrete et al., 2005; Negrete et al., 2006; Navaratnarajah et al., 2020). The soluble forms of HeV G protein was used to isolate 17 antibodies from a phage display human Fab naïve library (Zhu et al., 2006), to date the only report of phage display-derived anti-NiV/-HeV antibodies. Seven of them were further investigated and given the 80% sequence similarity between G proteins of HeV and NiV, some of them resulted in effective cross-recognition of the two viral species. Fab m102 was further improved through light chain shuffling, VH random mutagenesis and IgG1 conversion demonstrating cross-neutralization of both HeV and NiV and normal serum half-life in ferrets (Zhu et al., 2008). Further testing of this monoclonal antibody demonstrated its efficacy in NiV-infected ferrets (Bossart et al., 2009). For African green monkeys challenged with HeV (Bossart et al., 2011) and NiV (Geisbert et al., 2014), further evidence suggests that the therapeutic window in African green monkeys is different based on the NiV viral strain used (Mire et al., 2016). Its safety was also recently demonstrated in a Phase I first-in-human clinical trial (Playford et al., 2020), establishing the security criteria necessary to proceed to verify its effectiveness in infected humans in further clinical trials.

The Betacoronavirus genus (single-stranded, positive-sense RNA viruses) has clearly demonstrated its potential as source of worldwide health threats during the beginning of the new millennium, with SARS-CoV and SARS-CoV-2, both emerging from China in late 2002 and late 2019, respectively (Ksiazek et al., 2003; Lu et al., 2020). Furthermore, smaller outbreaks were caused by MERS virus in Saudi Arabia, during 2012 as it was first isolated, and South Korea (2015) (Zaki et al., 2012; Memish et al., 2020). Unlike the latter virus that keeps circulating in camelids (Sabir et al., 2016; Kandeil et al., 2019), SARS-CoV and SARS-CoV-2 originated from bats (Guan et al., 2003; Zhou et al., 2020) and spread worldwide. If the first SARS-CoV-caused pandemic was contained by summer 2003 (Cherry, 2004), COVID-19 pandemics is still lashing the entire world with recurring waves of infection. Phage display-derived antibodies are among the multiple approaches quickly deployed to generate monoclonal antibodies directed to SARS-CoV-2 (Jiang et al., 2020). In this emergence scenario, anti-nucleocapsid protein antibodies have been generated through phage display for diagnostic purposes (Kim et al., 2021). However, the most efforts have been focused on spike protein, and specifically to the receptor binding domain (RBD), to directly block the interaction with the host receptor, aiming to select neutralizing antibodies for novel therapeutics development. Various formats, e.g. IgG, Fab, VHH and bispecifics, from mainly human, either naïve or immune, libraries have been investigated and their neutralization efficiency was mostly studied *in vitro*, with some mAbs that were also tested in animal models (Li et al., 2020a; Li et al., 2020b; Noy-Porat et al., 2020; Sun Z. et al., 2020; Zeng et al., 2020; Bertoglio et al., 2021a; Bertoglio et al., 2021b; Bracken et al., 2021; Ku et al., 2021; Lim et al., 2021). At the moment of writing, the only anti-SARS-CoV-2 phage-display derived antibody known to enter clinical trials is STE90-C11 (COR-101) (ClinicalTrials.gov ID: NCT04674566) (Bertoglio et al., 2021a).

A summary of antibodies generated by phage display against viruses is presented in **Table 2**.

**Table 2:** Recombinant antibodies derived by phage display against viruses.

Virus	Target	Library Type	Antibody Format	Antibody Origin	Application	Reference
Australian bat Lyssavirus	possibly ABLV -G	naive	Fab, IgG	human	ELISA, *in vitro* neutralization	(Weir et al., 2021)
Avian influenza virus H7N2 (AIV)	complete virus	immune	VHH	camel	ELISA	(Gong et al., 2016)
Blue tongue virus	complete virus	semi-synthetic	scFv	chicken	ELISA	(van Wyngaardt et al., 2004; Fehrsen et al., 2005)
Blue tongue virus	complete virus	semi-synthetic	scFv, scFv-Fc	human	ELISA	(Ten Haaf et al., 2017)
Broad bean mottle virus (BBMV)	complete virus	immune	VHH	camel	ELISA, *in vitro* neutralization	(Ghannam et al., 2015)
Bovine viral diarrhea virus	nonstructural protein 5 (NS5B)	immune	VHH	camel	ELISA, intrabodies	(Duan et al., 2020)
Bovine viral diarrhea virus	envelope 2 (E2)	immune	VHH	camel	ELISA, *in vitro* neutralization	(Li et al., 2017)
Canine Parvovirus	VLP of CPV-VP2	immune	scFv	chicken	ELISA, WB, ICA, virus suppression assay	(Ge et al., 2020)
Cucumber mosaic cucumovirus	complete virus	semi-synthetic	scFv	human	ELISA, WB	(Ziegler et al., 1995)
Chikungunya virus (CHIKV)	VLPs	immune	VHH	llama	ELISA, *in vitro* neutralization	(Liu et al., 2019)
Chikungunya virus (CHIKV)	VLPs with E1 and E2	immune	Fab, IgG	human	ELISA, *in vitro* neutralization, *in vivo* protection	(Fong et al., 2014)
Dengue	envelope protein E	naïve	scFv	human	ELISA, Immunofluorescent assay, *in vitro* neutralization	(Saokaew et al., 2014)
Dengue	nonstructural protein 5 (NS5)	naïve	Fab	human	ELISA, WB, dot blot,	(Zhao et al., 2014)
Dengue	Dengue virus envelope protein	naïve	Fab, IgG	human,mouse (panel of hybridoma clones)	ELISA, WB, IHC	(Moreland et al., 2012)
Dengue	NS3	naïve	Fab	human	ELISA, *in vitro* neutralization	(Moreland et al., 2010)
Dengue	NS1	naïve(Non-immune)	VHH	llama	ELISA, lateral flow immunochromatograpic assay	(Fatima et al., 2014)
Dengue	n.d.	immune	Fab, IgG	chimpanzee	ELISA, immunoprecipitation, *in vitro* neutralization	(Men et al., 2004)
Dengue	NS1	immune	VHH	llama	ELISA (MagPlex assay)	(Shriver-Lake et al., 2018)
Dengue	NS1	naïve	scFv, Fab	human	ELISA, IF	(Lebani et al., 2017)
Dengue	NS1	naïve	scFv	human	ELISA, IF, *in vitro* neutralization	(Poungpair et al., 2014)
Dengue	DENV-2 RdRp	naive	scFv	human	ELISA, WB, *in vitro* inhibition	(Tunghirun et al., 2020)
Duck hepatitis A virus	VP1	immune	VHH	camel	ELISA, IF, dot-blot	(Xue et al., 2019)
Duck hepatitis A virus	VP3	immune	scFv	mice	ELISA, *in vivo* neutralization	(Wang et al., 2018)
Ebola	nucleoprotein, envelope glycoprotein, secreted envelope glycoprotein	immune	Fab, IgG	human	ELISA, immunostaining, immunoprecipitation, *in vitro* neutralization	(Maruyama et al., 1999a)
Ebola	nucleoprotein	synthetic	scFv, IgG	human	ELISA, WB	(Shingarova et al., 2007)
Ebola (Zaire)	viral matrix protein VP40, nucleoprotein	immune	scFv, IgNAR	mice, shark	ELISA, WB	(Goodchild et al., 2011)
Ebola (Zaire)	VP35	semi-synthetic	scFv	human	ELISA, WB, intrabody	(Flego et al., 2019)
Ebola (Sudan)	glycoprotein (GP)	immune	scFv, scFv-Fc	macaque	ELISA, *in vitro* neutralization, *in vivo* protection	(Froude et al., 2018; Herbert et al., 2020)
Ebola (Zaire)	glycoprotein (GP)	immune	VHH	llama	ELISA	(Liu et al., 2017)
Ebola (Zaire)	VP35, interferon inhibitory domain	naïve	scFv (transbody)	human	ELISA, IF, WB	(Seesuay et al., 2018)
Ectromelia virus (ECTV)	ECTV, variola virus, vaccinia virus, cow pox virus	immune	scFv	human	ELISA, *in vitro* neutralization, WB	(Khlusevich et al., 2018)
Enterovirus 71 (EV71)	virion protein 2 (VP2)	naïve	scFv	mice	ELISA, WB	(Thanongsaksrikul et al., 2018)
Enterovirus 71	inactivated EV71 virions	immune	Fab	human	ELISA, *in vitro* neutralization	(Chen et al., 2017)
Enterovirus 71/coxsackievirus A16 (CVA16)	VP4	immune	scFv, IgG	mice	WB, *in vitro* neutralization	(Zhang et al., 2016)
Enterovirus 71	VP4	naïve	scFv	human	ELISA, WB, IF, *in vitro* neutralization	(Phanthong et al., 2020)
Epstein-Barr virus	LMP1	naïve	Fab	human	ELISA, WB, IF, FACS, *in vitro* inhibition	(Zhang et al., 2013)
Foot-and-mouth disease virus (FMDV)	3ABC	immune	scFv	chicken	ELISA, WB	(Foord et al., 2007)
Foot-and-mouth disease virus	146S	immune	VHH	llama	ELISA	(Harmsen et al., 2017)
Foot-and-mouth disease virus	VP2 capsid protein	immune	scFv	mice	ELISA	(Salem et al., 2019)
Foot-and-mouth disease virus	VLPs	immune	VHH	camel	ELISA, IF	(Wang D. et al., 2015)
Grapevine leafroll-associated virus 3	coat protein	immune	scFv	mice	ELISA	(Orecchia et al., 2008)
Grapevine virus B	virus particles	semi-synthetic	scFv	human	ELISA	(Saldarelli et al., 2005)
Haematopoietic necrosis virus (IHNV)	n.d.	naïve(non-immune)	scFv	mouse	ELISA, WB, IHC	(Liu H. et al., 2014)
Hantavirus	nucleoprotein	immune	VHH	llama	ELISA, WB	(Pereira et al., 2014)
Hantavirus	virus particles	immune	Fab	human	IF, WB	(Koch et al., 2003)
HCMV	gycoprotein B and H	immune	scFv	human	ELISA, *in vitro* neutralization	(Nejatollahi et al., 2002)
HCMV	Gp55	immune	scFv	human	ELISA, *in vitro* neutralization	(Moazen et al., 2016)
Hendra and Nipah virus	attachment envelope glycoprotein G	naïve	Fab, IgG	human	ELISA, immunoprecipitation, WB, *in vitro* neutralization IF, *in vivo* neutralization	(Zhu et al., 2006; Zhu et al., 2008)
Hepatitis A	Hepatitis A Capsid	immune	Fab, IgG	chimpanzee	ELISA, *in vitro* neutralization	(Schofield et al., 2002)
Hepatitis A	HBsAG	naïve	scFv	human	ELISA	(Zhang J.-L et al., 2006)
Hepatitis C	NS5B	naïve	scFv	human	ELISA, IF, *in vitro* neutralization	(Thueng-in et al., 2014)
Hepatitis C	core, E1, E2	immune	scFv	human	ELISA	(Chan et al., 1996)
Hepatitis C	core protein	immune	Fab	human	ELISA	(Barban et al., 2000)
Hepatitis C	E2 glycoprotein	immune	Fab	human	ELISA	(Burioni et al., 2001)
Hepatitis C	NS5A	naïve	scFv (transbodies)	human	ELISA, WB, IF, *in vitro* neutralization	(Glab-Ampai et al., 2017)
Hepatitis C	NS3/4A	naïve	scFv (transbodies)	human	ELISA, IF, *in vitro* neutralization	(Jittavisutthikul et al., 2016)
Hepatitis E	ORF2 protein	immune	Fab	chimpanzee	ELISA, WB	(Schofield et al., 2000)
Herpes simplex virus	HSV1, -2 lysate	immune	Fab	human	*in vitro* inhibition, immuno precipitaton	(Burioni et al., 1994)
Herpes simplex virus	glycoprotein gD, gB	presumably immune	Fab	human	ELISA, immuno precipitation	(Sanna et al., 1995)
Herpes simplex virus	virus lysate?	immune	Fab	human	ELISA, IF	(Cattani et al., 1997)
HIV-1	Integrase	immune	scFv	rabbit	ELISA, WB, IF, *in vitro* neutralization	(da Silva et al., 2012)
HIV-1	gp140	immune	scFv, scFv-Fc	human	ELISA, WB, immunoprecipitation, *in vitro* neutralization	(Trott et al., 2014)
HIV-1	gp140	immune	Fab, IgG	human	ELISA, WB, *in vitro* neutralization	(Choudhry et al., 2007)
HIV-1	gp140	immune	VHH	llama	ELISA, *in vitro* neutralization	(Strokappe et al., 2012)
HIV-1	gp120	synthetic	CH2 domain	human	ELISA, *in vitro* neutralization	(Xiao et al., 2009)
HIV-1	p24	immune	scFv	mouse	ELISA	(Mohammadzadeh et al., 2014)
HIV-1	gp41	synthetic	Fab	human	WB, *in vitro* neutralization	(Gustchina et al., 2007)
HIV-1	gp41	naïve	scFv	human	ELISA, *in vitro* neutralization	(Miller et al., 2005)
HIV-1	gp120	immune	Fab	human	ELISA, *in vitro* neutralization, IF	(Sun et al., 2017)
HIV-1	gp140	immune	VHH	camel	ELISA, *in vitro* neutralization	(Koch et al., 2017)
HIV-1	gp140 trimer	immune	scFv	human	ELISA, *in vitro* neutralization	(He et al., 2017)
HIV-1	RSC3 core protein	immune	scFv	human	ELISA, *in vitro* neutralization	(Kumar et al., 2017)
HIV-2	gp125 protein	immune	Fab	human	ELISA, *in vitro* neutralization	(Björling et al., 1999)
Human metapneumovirus	F ectodomain	presumably immune	Fab	human	ELISA, IF, *in vitro* neutralization, *in vivo* protection	(Williams et al., 2007)
Influenza A (IAV)	HA (stem region)	semi- synthetic	scFv	human(IGHV1-69)	ELISA, in *vitro neutralization*	(Avnir et al., 2014)
Influenza A	HA ectodomain	naïve	scFv	human	ELISA, Flow cytometry, immunoprecipitation, *in vitro* neutralization, *in vivo* protection	(Sui et al., 2009)
Influenza A (H2N2)	HA (stem region)	presumably immune	Fab	human	ELISA, *in vitro* neutralization	(Wyrzucki et al., 2014)
Influenza A	HA2	semi-synthetic	scFv	human	ELISA, WB, *in vitro* neutralization	(Li et al., 2016)
Influenza A (H1N1)	HA	immune	scFv	mice	ELISA	(Rajput et al., 2015)
Influenza A (H1N1/H5N1)	HA	immune	VHH, bivalent VHH	alpaca	ELISA, *in vitro* neutralization	(Hufton et al., 2014)
Influenza A (H5N1)	HA	semi-synthetic	scFv	human	ELISA	(Wu et al., 2014)
Influenza A (H5N1)	HA	naïve	scFv	human	ELISA, *in vitro* neutralization, *in vivo* protection	(Maneewatch et al., 2009)
Influenza A (H5N1)	HA	immune	Fab	chicken	IF, *in vitro* neutralization, WB	(Pitaksajjakul et al., 2010)
Influenza A (H5N1)	nucleoprotein (NP)	immune	scFv	mice	ELISA, WB, *in vitro* inhibition	(Sengupta et al., 2014)
Influenza A (H5N1)	HAcleavage site	immune	Fab	mice	ELISA, IF	(Dong et al., 2013)
Influenza A (H5N1)	NS1	naïve	scFv	human	ELISA, *in vitro* neutralization, IF	(Yodsheewan et al., 2013)
Influenza A (H5N1)	complete inactivated virus	immune	VHH	camel	ELISA	(Zhu et al., 2014)
Influenza A (H3N2)	peptides (neutralizing epitope)	semi-synthetic	scFv	human	ELISA	(Vashisht et al., 2019)
Influenza A	M2-cytoplasmatic domain	naïve	scFvscFv-Fc	human	WB, IHC	(Velappan et al., 2020)
Influenza B	HA	immune	VHH	alpaca	ELISA	(Ramage et al., 2019)
Influenza B	whole virus	immune	Fab	human	ELISA, WB, IF, *in vitro* neutralization	(Hirano et al., 2018)
Japanese encephalitis virus	domains I,II,III of envelope protein	immune	Fab, IgG	chimpanzee	ELISA, immunoprecipitation, *in vitro* neutralization, *in vivo* protection	(Goncalvez et al., 2008)
Japanese encephalitis virus	envelope Protein	immune	Fab	human	ELISA, immunoprecipitation, *in vitro* neutralization	(Arakawa et al., 2007)
Marburg virus	glycoprotein (GP)	immune	scFv, scFv-Fc	macaque	WB, *in vitro* neutralization, *in vivo* protection	(Froude et al., 2017)
Marburg virus	VP35	synthetic	Fab	human	ELISA	(Amatya et al., 2019)
Measles virus	virus lysate	immune (Puumala hantavirus)	Fab	human	ELISA, *in vitro* neutralization	(de Carvalho Nicacio et al., 2002)
MERS	S2 domain	synthetic	Fab, IgG	human	ELISA, IF, sandwich ELISA	(Kim et al., 2019)
MERS	nucleoprotein (NP)	naïve	scFv	human	ELISA	(Lim et al., 2019)
Norovirus	Norovirus VLPs	semi-synthetic	scFv	human	ELISA, WB	(Huang et al., 2014)
Norovirus	P-domain of the GI.1 major capsid protein	semi-synthetic	scFv	human	ELISA, dot-blot	(Hurwitz et al., 2017)
Norovirus	VLPs with VP1 and VP2	immune	VHH	llama	ELISA, WB, *in vitro* inhibition, IF	(Garaicoechea et al., 2015)
Norovirus	VLPs	naïve	scFv	human	ELISA, *in vitro* inhibition	(Higo-Moriguchi et al., 2014)
Norwalk virus	VLPs	immune	Fab, IgG	chimpanzee	ELISA, FACS, IF, *in vitro* inhibition	(Chen et al., 2013)
Paramyxovirus	glycoproteins F and HN	synthetic	Fab, sAb	human	ELISA, *in vitro* neutralization, immunoprecipitation	(Welch et al., 2014)
Plum pox virus	NIa protease	semi-synthetic	scFv	human	WB, dotblot	(Hust et al., 2002)
Poliovirus	capsid proteins VP1 and VP3	immune	Fab, IgG	chimpanzee	ELISA, *in vitro* neutralization, *in vivo* protection	(Chen et al., 2011)
Polyomavirus JC (JCV)	VLPs	synthetic	Fab, IgG	human	ELISA	(Chen et al., 2015)
Porcine circovirus type 2	Cap protein	immune	VHH	camel	ELISA	(Mu et al., 2021)
Porcine epidemic diarrhea virus	PEDV S1 domain of spike protein	immune	VHH	camel	ELISA, IF	(Bao et al., 2019)
Porcine epidemic diarrhea virus	nucleocapsid (N) protein	immune	VHH	camel	ELISA	(Ma et al., 2019)
Porcine epidemic diarrhea virus	spike protein	immune	scFv	pig	ELISA, IF, *in vivo* protection	(Zhang et al., 2019a)
Porcine reproductive and respiratory syndrome virus (PRRSV)	non-structural protein 4 (NSP4)	immune	VHH (intrabody)	camel	ELISA, IF, *in vitro* neutralization	(Liu et al., 2016)
Porcine reproductive and respiratory syndrome virus (PRRSV)	Nsp9	iummune	VHH	camel	ELISA, IF, *in vitro* inhibition	(Liu et al., 2015)
Puumala hantavirus	N protein, G2 protein	immune	Fab	human	ELISA	(Salonen et al., 1998)
Puumala hantavirus	gycoprotein G2	immune	Fab	human	ELISA, IF, *in vitro* neutralization	(de Carvalho Nicacio et al., 2000)
Rabies virus	glycoprotein	semi-synthetic	scFvscFv-Fc	human	ELISA, WB, *in vitro* neutralization	(Ray et al., 2001)
Rabies virus	glycoprotein	immune	scFv, IgG	human	ELISA, flow cytometry, *in vitro* neutralization	(Bakker et al., 2005; Kramer et al., 2005)
Rabies virus	n.d.	immune	Fab	human	ELISA	(Houimel, 2014)
Rabies virus	glycoprotein (antigenic site II)	immune	Fab, IgG	human	ELISA, immunostaining, WB, *in vitro* neutralization, *in vivo* protection	(Sun et al., 2012)
Rabies virus	inactivated RABV	naïve	VHH, VHH pentamer	lama	ELISA, *in vitro* neutralization, *in vivo* protection	(Boruah et al., 2013)
Respiratory syncytial virus (RSV)	F protein	synthetic	Fab, IgG	human	ELISA, *in vitro* neutralization	(Chen et al., 2016)
Rotavirus	NSP4	semi-synthetic	scFv	human	ELISA, WB	(Rodríguez-Díaz et al., 2004)
Rotavirus	VP8*	semi-synthetic	scFv	human	ELISA, WB, *in vitro* inhibition	(Monedero et al., 2004)
SARS-CoV	S1 domain of spike protein	naïve	scFv	human	ELISA, *in vitro* neutralization	(Sui et al., 2004)
SARS-CoV	S protein	immune	Fab, IgG	human	ELISA, IF, immuno blot, *in vitro* neutralization	(Liang et al., 2005)
SARS-CoV	S protein	immune	scFv	chicken	ELISA, IF	(Lee et al., 2007)
SARS-CoV-2	S protein (RBD)	naïve	scFv, scFv-Fc, IgG	human	ELISA, *in vitro* inhibition, *in vitro* neutralization	(Bertoglio et al., 2021b)
SARS-CoV-2	S protein (RBD)	immune	scFv, scFv-Fc, IgG	human	ELISA, *in vitro* inhibition, *in vitro* neutralization, *in vivo* protection	(Bertoglio et al., 2021a)
SARS-CoV-2	S protein (RBD)	naïve	scFv, scFv-Fc	human	ELISA, *in vitro* neutralization	(Noy-Porat et al., 2020)
SARS-CoV-2	S protein (RBD)	synthetic	VHH	humanized	*in vitro* neutralization	(Chi et al., 2020)
SARS-CoV-2	S protein (RBD)	synthetic	Fab	human	ELISA, *in vitro* neutralization	(Zeng et al., 2020)
SARS-CoV-2	S protein (RBD)	immune	VHH, VHH-Fc	alpaca	ELISA, *in vitro* inhibition, IF	(Ma et al., 2021)
SARS-CoV-2	S protein (RBD)	semi-synthetic	scFv, scFv-Fc, IgG	human	ELISA, WB	(Parray et al., 2020)
SARS-CoV-2	S protein (RBD)	immune	VH	human	ELISA, FACS, *in vitro* neutralization	(Sun et al., 2020)
SARS-CoV-2	S protein (RBD)	synthetic	VH	human	ELISA, *in vitro* neutralization	(Bracken et al., 2021)
SARS-CoV-2	S protein (RBD)	synthetic	Fab, bispecific Fab+VH	human	*in vitro* neutralization	(Lim et al., 2021)
SARS-CoV-2	S protein (RBD)	naïve	scFv	human	ELISA, *in vitro* neutralization, *in vitro* inhibition, *in vivo* protection	(Ku et al., 2021)
SARS-CoV-2	RBD	immune	Fab, IgG	human	ELISA, *in vitro* neutralization assay,*in vivo* protection,	(Zhou et al., 2021)
SARS-CoV-2	RBD	semi-synthetic	Fab	human	ELISA, *in vitro* neutralization	(Slezak and Kossiakoff, 2021)
SARS-CoV-2	RBD	immune	VHH	alpaca	ELISA, *in vitro* inhibition, *in vitro* neutralization	(Gai et al., 2021)
SARS-CoV-2	NP	immune	scFv, scFv-Fc	chicken	ELISA, dotblot, lateral flow strip assay	(Kim et al., 2021)
SARS-CoV-2	NP	immune	Fab, IgG	human	ELISA, WB	(Zhang et al., 2020)
Simian immunodeficiency virus (SIV)	gp120	immune	Fab	rhesus macaque	ELISA, *in vitro* neutralization	(Glamann et al., 1998)
Simian immunodeficiency virus (SIV)	gp120/gp140	immune	scFv, scFv-Fc	rhesus macaque	WB, *in vitro* neutralization	(Ita et al., 2017)
Sin Nombre Virus	SNV-N	naïve	scFv	human	ELISA, WB, dot-blot	(Velappan et al., 2007)
Swine Influenza Virus	SIV-NP	immune	VHH	camel	ELISA, WB	(Du et al., 2019)
Transmissible gastroenteritis virus	TGEV whole virus	immune	scFv	pig	*in vitro* neutralization, IF, WB	(Zhang et al., 2019b)
Usutu virus	USUV A, USUV B	immune	scFv	chicken	ELISA, WB, *in vitro* neutralization	(Schoenenwald et al., 2020)
Vaccinia virus, Variola Virus	Vaccinia B5 envelope protein	immune	Fab, IgG	chimpanzee	ELISA, *in vitro* neutralization, *in vivo* protection	(Chen et al., 2006)
Vaccinia virus	virus particles	immune	scFv, IgG	human	inhibition ELISA, *in vitro* neutralization	(Shin et al., 2019)
Vaccinia virus	inactivated virus particles, L1	immune	VHH	llama	MAGPIX assay, FACS	(Walper et al., 2014)
VEEV	virus particles	immune	scFv	mice	ELISA	(Duggan et al., 2001)
VEEV	E1/E2	naïve	scFv, scFv-Fc	human	ELISA, WB, IHC	(Kirsch et al., 2008)
VEEV	E1	immune	scFv, scFv-Fc	macaque	ELISA, WB, IHC, *in vitro* neutralization, *in vivo* protection	(Rülker et al., 2012)
WEEV	virus particles	immune	scFv, scFv-Fc	macaque	ELISA, IHC, *in vitro* neutralization, *in vivo* protection	(Hülseweh et al., 2014; Burke et al., 2018)
WEEV	E2/E3E2	immune	VHH	llama	ELISA (MagPlex assay)	(Liu et al., 2018)
West nile virus	domain I and II of WNV envelope protein	naïve	scFv	human	ELISA, *in vitro* neutralization, *in vivo* protection	(Gould et al., 2005)
West nile virus	domain IIII of WNV envelope protein	immune	Fab	human	ELISA, IF, *in vitro* neutralization, *in vivo* protection (failed)	(Duan et al., 2009)
West nile virus	envelope (E)	naïve	scFv, scFv-Fc	human	ELISA, WB, *in vitro* neutralization	(Rizzo et al., 2020)
White spot syndrome virus	virus particles	immune	scFv	mice	ELISA, *in vitro* neutralization	(Yuan et al., 2006)
Yellow fever virus	domain II of envelope protein	immune	scFv, IgG	human	ELISA,WB, immunoprecipitation, *in vitro* neutralization, *in vivo* protection	(Daffis et al., 2005; Lu et al., 2019)
Zika virus	envelope domain III	naïve	Fab	human	*in vitro* neutralization, *in vivo* protection	(Wu et al., 2017)
Zika virus	envelope (E)	immune	scFv	chicken	ELISA, WB, FACS, *in vitro* inhibition	(Mwale et al., 2020)
Zika virus	NS1	Immune	VHH	llama	ELISA	(Delfin-Riela et al., 2020)

ELISA, enzyme linked immunosorbent assays; HA, hemagglutinin; HCMV, Human cytomegalovirus; HIV, Human immunodeficiency virus; IF, immuno fluorescence microscopy; IHC, immunohistochemistry; RABV, Rabies virus; MERS, Middle East Respiratory Syndrome Coronavirus; SARS, Severe acute respiratory syndrome; VLP, virus like particle; VEEV, Venezuelan equine encephalitis virus; WB, western blot; WEEV, Western equine encephalitis virus.

## Eukaryotic Pathogens

A huge number of antibodies against a broad range of eukaryotic pathogens has been generated by phage display. These recombinant antibodies are directed against very different parasites, e.g. *Taenia solium* (Ribeiro et al., 2013), protozoa, e.g. *Cryptosporidium parvum* (Boulter-Bitzer et al., 2009; Boulter-Bitzer et al., 2010), *Plasmodium falciparum* (Roeffen et al., 2001; Lundquist et al., 2006) or *Toxoplasma gondii* (Hoe et al., 2005) and fungi such as *Aspergillus fumigatus* (Schütte et al., 2009). Beside human pathogens also veterinary pathogens like *Myxobolus rotundus* (Zhang J. Y. et al., 2006) (a fish pathogen) or *Babesia gibsoni* (a dog pathogen) (Hirose et al., 2009) and plant pathogens like *Aspergillus niger* (Ascione et al., 2004), *Fusarium verticilloides* (Hu et al., 2012) or *Sclerotinia sclerotiorum* (Yang et al., 2009) are of interest.

The majority of the antibodies to these targets are derived from human antibody gene libraries, but libraries derived also from mouse (Fu et al., 1997), chicken (Hu et al., 2012), camel (Saerens et al., 2008) or macaque (Schütte et al., 2009) have been successfully applied to select recombinant antibodies against eukaryotic pathogens.

Detailed examples for the recombinant antibodies against several eukaryotic pathogens derived from phage display are given the following paragraphs.

Allergic bronchopulmonary aspergillosis, saprophytic aspergilloma, chronic necrotizing aspergillosis and the invasive aspergillosis which is highly lethal are caused by *Aspergillus fumigatus* and are the most important *Aspergillus*-related diseases (Broderick et al., 1996; Latgé, 1999). As one possible consequence of a hematopoietic stem cell transplantation or solid organ transplantation, an invasive aspergillosis can occur in these immunocompromised patients (Rüping et al., 2008). Therefore, an early diagnosis of aspergillosis is crucial for a successful treatment. The development of a panel of human antibodies binding to Crf2 was described by Schütte et al. (2009). The glycosyol hydrolase Crf2 is located in the cell wall of the growing hyphae. Two kind of antibody gene libraries were used: a macaque immune library and the human naïve antibody libraries HAL4/7 (Hust et al., 2011). In addition, two different antibody selection strategies were performed: A) a panning on recombinant antigen immobilized on immunostrips. B) a panning in solution using biotinylated antigen. Six antibodies were selected from the human naïve libraries and ten from the macaque immune library. It was observed, that all antibodies generated by panning in solution bound to conformational epitopes and the antibodies generated on directly immobilized Crf2 bound to linear epitopes. Seven antibodies bound to the native antigen Crf2 on growing hyphae of *Aspergillus fumigatus* shown by histopathological immunofluorescence microscopy. These antibodies didn’t show cross reactions with other *Aspergillus* species or *Candida albicans*. It was shown that anti-Crf2 antibody MS112-IIB1 was able to neutralize the enzymatic activity of Crf2, but was not able to reduce the fungal growth in a rat model of invasive pulmonary aspergillosis (Chauvin et al., 2019).

Besides the diagnosis in patients, the preventive diagnosis of Aspergillus pathogens in agricultural products is of importance. Therefore, Wang et al. developed a nanobody-polyclonal antibody Sandwich-ELISA (Wang T. et al., 2017). To obtain polyclonal antibodies and for the construction of a camelid immune library, rabbits or alpacas, respectively, were immunized with extracellular antigens and mycelia lysate of A. flavus, a strain with high levels of aflatoxin. Panning resulted in one VHH nanobody against the extracellular antigens and two nanobodies against the mycelia lysate. The developed Sandwich-ELISA is detecting aflatoxin producing strains of the *Aspergillus* spp. in agricultural products before the fungi can produce high levels of aflatoxin and is therefore suited for early detection.

Malaria is one of the most common mosquito-borne diseases caused by five different species of *Plasmodium*, *P. falciparum*, *P. vivax*, *P. malariae, P. ovale* and *P. knowlesi* (Cox-Singh et al., 2008). In humans *P. falciparum* is responsible for most malaria-related deaths globally, while *P. vivax* is the most widespread parasite (Popovici and Ménard, 2015). The parasites infect erythrocytes and destroy these cells at the end of the replication cycle. In tropical and subtropical countries there are approximately 3.2 billion people at risk of malaria (Tolle, 2009; Muerhoff et al., 2010). Because the life cycle of *Plasmodium* consists of different development stages, different targets of *P. falciparum* were addressed for antibody generation. Roeffen et al. (2001) targeted Pfs48/45, a surface protein of *P. falciparum* which is expressed during macrogamete and zygote stages. Pfs48/45 is also a potential vaccine candidate because transmission-blocking anti-Pfs48/45 antibodies taken up by the mosquito during the blood meal and block the oocyte development within the mosquito’s intestinal tract (Rener et al., 1983; Kumar and Carter, 1984; Vermeulen et al., 1985). To generate anti-Pfs48/45 antibodies, two human immune libraries were constructed from B-lymphocytes derived from *P. falciparum* patients with transmission-blocking immunity.

The antibody selection was performed in immunotubes with immobilized extract of gametocytes. To specifically generate antibodies against Pfs48/45, the scFv-phage were eluted by competition using a cocktail of four rat monoclonal Abs recognizing distinct epitopes on Pfs48/45 (epitopes I, IIb, III and V). Remarkably, one epitope, the epitope III of Pfs48/45, was bound by all of the generated antibodies. Human monoclonal antibodies against the Block 2 region of *Plasmodium falciparum* merozoite surface protein-1 (PfMSP-1) were isolated by phage display from a scFv immune library of a malaria patient (Sowa et al., 2001). A Fab-immune library was constructed using lymphocytes of thirteen adults with acquired immunity to malaria. Finally, three Fabs (RAM1, 2 and 3) were isolated by panning on the merozite surface protein 3 (MSP-3_194-257_) (Lundquist et al., 2006). This protein is involved in the binding of heme and the antibodies promote the depletion of *Plasmodium* by monocytes (Oeuvray et al., 1994; Imam et al., 2014). Therefore, the N-terminal fragment of MSP-3 was also tested in clinical vaccines trials (Audran et al., 2005). Binding of the antibodies to native parasite protein was demonstrated for all three antibodies in immunofluorescence microscopy and immunoblot. The native MSP-3 was bound by RAM1 and RAM2 in fixed and permeabilized cells. The anti-MSP-3 antibodies were produced as IgG1 and also in the IgG3 format and tested in an antibody-dependent cellular inhibition assay (ADCI). The IgG3 format of RAM1 showed an inhibition rate that is comparable to affinity-purified polyclonal anti-MSP-3_211-237_ antibodies derived from human immune donors. In contrast, the IgG1 format also showed a lower inhibition in this assay compared to IgG3 (Lundquist et al., 2006). In 2021, Seidel-Greven et al. (2021) presented a monoclonal antibody directed against AMA-1, the apical membrane antigen 1. As anti-AMA-1 antibodies show a high prevalence in immune individuals it is not only an interesting target for therapeutics but also one of the major blood-stage vaccine candidates. A human malaria Fab-immune library was used to select antibodies against a set of three AMA-1 variants. To improve the affinity and functional activity a light chain shuffling was performed. After subcloning to IgG1/ϰ, affinity and competition studies were performed by surface plasmon resonance (SPR) spectroscopy. In addition to an immunofluorescence assay (IFA), the antibodies were tested in an *in vitro* growth assay. Here, all antibodies were able to inhibit the growth of four different *P. falciparum* strains including 3D7. Therefore, those antibodies might be the fundament for the development of an antibody cocktail for therapeutic or prophylactic anti-malaria approaches. In 2014, Leow et al. (2014) aimed to find new antibodies with higher thermal stability. As degradation of antibodies due to high temperatures in the endemic countries are affecting the shelf life of malaria rapid diagnostic tests (RDTs). Therefore, they performed a panning with a naïve human scFv library on the histidine-rich protein 2 (PfHRP2) of *P. falciparum*. PfHRP2 is a biomarker used in most RDTs, as it is abundantly expressed in erythrocytes across all asexual stages and is released during the rupture of the red blood cells. Additionally, it remains detectable in the blood for up to 28 days. Isolated binders were cloned into the IgG format, produced in CHO cells and were compared to a commercial antibody. The selected antibodies showed comparable specificity and sensitivity, with higher thermal stability, making them candidates for a field test in a prototype test kit. To further cope with the degradation issues, Leow et al. (2018) created a shark immune library consisting of VNARs. The variable domain of the shark antibodies is known to have a high thermal stability and therefore is a potential alternative for the RDTs. A wobbegong shark (Orectolobus ornatus) was immunized with different malaria proteins (PfHRP2, PfpLDH and Pvaldolase), used for the construction of a VNAR single domain library and antibodies were selected against all three proteins. Pvaldolase and pLDH are used for diagnosis of non *P. falciparum* malaria infections and *P. vivax* infections. Specificity and sensitivity of the anti-PfHRP2 antibodies were demonstrated by Sandwich-ELISA and dot blot analysis. Further engineering could lead to a new generation of RDTs.

**Table 3** gives an overview of antibodies generated by phage display against eukaryotic pathogens.

**Table 3:** Recombinant antibodies derived by phage display against eukaryotic pathogens.

Eukaryotic pathogen	Target	Library Type	Antibody Format	Antibody Origin	Application	Reference
*Alexandrium minutum*	Whole cell/surface epitopes	naïve	nanobody/fluobody	lama	IF	(Mazzega et al., 2019)
*Aspergillus flavus*	extracellular antigens & mycelia lysate	immune	nanobody	alpaca	ELISA (sandwich), WB	(Wang T. et al., 2017)
*Aspergillus fumigatus*	Crf2	immune/naïve	scFv,scFv-Fc	macaque/human	ELISA, IF	(Schütte et al., 2009)
*Aspergillus niger*	glucose oxidase	semi-synthetic	scFv	human	ELISA	(Ascione et al., 2004)
*Babesia gibsoni*	P50	semi-synthetic	scFv	human	ELISA, IF	(Hirose et al., 2009)
*Balamuthia mandrillaris*	whole cell/surface antigens	naïve	scFv	human	ELISA, IHC	(Siddiqui et al., 2016)
*Botrytis cinerea*	fungal glucosylceramides (fGlcCer)	immune	VHH	llama	ELISA, *in vitro* activity assay	(De Coninck et al., 2017)
*Brugia malayi*	BmR1	naïve, immune	scFv	human	ELISA, WB	(Rahumatullah et al., 2017)
*Brugia malayi*	BmSXP	immune	scFv	human	ELISA, WB	(Rahumatullah et al., 2015; Rahumatullah et al., 2019)
*Candida albicans*	Als3p and other	naïve	scFv	human	ELISA, IF, WB, *in vitro* neutralization	(Haidaris et al., 2001; Bliss et al., 2003; Laforce-Nesbitt et al., 2008)
*Cryptosporidium parvum*	P23	semi-synthetic	scFv	human	ELISA	(Boulter-Bitzer et al., 2009)
*Cryptosporidium parvum*	S16	semi-synthetic	scFv	human	ELISA	(Boulter-Bitzer et al., 2010)
*Echinococcus granulosus*	AgB	immune	scFv	human	ELISA	(Rahumatullah et al., 2020)
*Fasciola hepaticum*	cathepsin L1	immune	scFv	rat	ELISA, WB	(Norbury et al., 2019a)
*Fasciola hepaticum*	cathepsin B2	naïve	scFv	mice	ELISA, WB	(Norbury et al., 2019b)
*Fusarium* oxysporum	cell wall-bound protein	immune	scFv	chicken	ELISA, IF, scFv-AFP (anti-fungal peptide) fusion protein	(Peschen et al., 2004)
*Fusarium verticillioides*	cell wall-bound proteins	immune	scFv	chicken	ELISA, IF	(Hu et al., 2012)
*Fusarium verticillioides*	soluble cell wall-bound proteins	immune	scFv,scFv-AP	chicken	ELISA, IF, WB	(Hu et al., 2013)
*Haemonchus contortus*	excretory/secretory (ES) products	immune	scAb	sheep	IF, transepithelial resistance assay	(Rehman et al., 2016)
*Leishmania infantum*	β-tubulin	naïve	scFv	human	ELISA, WB	(Costa et al., 2019)
*Myxobolus rotundus*	spore protein	immune	scFv	mice	ELISA, *in vitro* neutralization	(Zhang J. Y. et al., 2006)
*Neospora caninum*	rNcSRS2	immune	Fab	mice	ELISA, IF	(Dong et al., 2014)
*Onchocerca volvulus*	Ov16	synthetic	Fab, IgG	human	ELISA, rapid diagnostic platform	(Golden et al., 2016)
*Paranosema locustae*	alpha-/beta-hydrolase	immune	scFv, mini antibody	mice	WB, IF	(Dolgikh et al., 2017)
*Plasmodium chabaudi*	AMA-1	immune	scFv	mice	ELISA, WB	(Fu et al., 1997)
*Plasmodium falciparum*	MSP-1	immune	scFv	human	ELISA, IF	(Sowa et al., 2001)
*Plasmodium falciparum*	MSP-3	immune	Fab,IgG	human	ELISA, IF, WB, FACS	(Lundquist et al., 2006)
*Plasmodium falciparum*	Pfs48/45	immune	scFv	human	ELISA, WB	(Roeffen et al., 2001)
*Plasmodium falciparum*	PfHRP2, PfpLDH and Pvaldolase	Immune/sdAb	VNAR	shark	ELISA, dot blot	(Leow et al., 2018)
*Plasmodium falciparum*	PfHRP2	naïve	scFv	human	ELISA (sandwich), WB, dot blot	(Leow et al., 2014)
*Plasmodium falciparum*	AMA-1	immune	Fab, IgG	human	ELISA, IF, *in vitro* inhibition	(Seidel-Greven et al., 2021)
*Plasmodium yoelii*	MSP1	immune	scFv	mice	ELISA, WB, *in vivo* protection	(Vukovic et al., 2002)
*Schisostoma japonicum*	*Schisostoma japonicum* cercariaeschistosomules	immune	scFv	buffalo	IF, WB, ELISA, protein microarray	(Hosking et al., 2015a; Hosking et al., 2015b)
*Schizophyllum commune*	beta-(1,6)-Branched beta-(1,3)-D-Glucan Schizophyllan	immune	scFv	mice	ELISA	(Josewski et al., 2017)
*Strongyloides stercoralis*	rNIE	immune	scFv	human	ELISA, WB	(Rahumatullah et al., 2021)
*Sclerotinia sclerotiorum*	SSPG1d	immune	scFv	mice	ELISA, WB	(Yang et al., 2009)
*Strongyloides venezuelensis*	HSP60	presumably naïve	scFv	human	ELISA, IF	(Levenhagen et al., 2015)
Taenia solium	TS14	immune	VHH	camel	ELISA, WB	(Deckers et al., 2009)
Taenia solium	*T. solium* metacestodes, peptides	naïve	scFv	human	ELISA, IF	(Ribeiro et al., 2013)
*Trypanosoma cruzi*	P2ß	immune	scFv	human	ELISA, WB	(Grippo et al., 2011)
*Trypanosoma evansi*	different surface proteins	immune	VHH	camel	ELISA, FACS	(Saerens et al., 2008)
*Trypanosoma evansi*, *brucei, T. congolense,T. vivax*	different surface proteins	immune	VHH	alpaca	WB, IHC	(Obishakin et al., 2014)
*Toxoplasma gondii*	TgMIC2	immune	scFv	mice	ELISA, WB	(Hoe et al., 2005)
*Toxoplasma gondii*	tachyzoites	immune	scFv	mice	ELISA, WB, IF, *in vitro* invasion, attachment assays	(Lim et al., 2018)

ELISA, enzyme linked immunosorbent assays; IHC, immuno histo chemistry; IF, immuno fluorescence microscopy; FACS, fluorescence-activated cell sorting; WB, Western blot.

## Antibodies Against Toxins

The Center for Disease Control and Prevention (CDC) classifies several bacterial toxins and/or the pathogens producing these toxins as category A or B agent as they are a high risk to national security and public health (see: https://www.niaid.nih.gov/research/emerging-infectious-diseases-pathogens). Thus, these toxins are a relevant target for development of diagnostics and therapeutics (Froude et al., 2011). Here, antibody phage display technology is a powerful tool to select diagnostic as well as neutralizing antibodies against complete active toxins or certain domains by using human naïve antibody libraries with high diversity (Indrawattana et al., 2010; Amaro et al., 2011; Neri et al., 2011). The aim of such neutralizing antibodies is in most cases to block the interaction of the toxin to its cellular target, by binding to the cell binding domain of the toxin. However, neutralization of the toxicity is also possible by antibodies directed against other domains like the translocation domain or the enzymatic domain (Fühner et al., 2018; Wenzel et al., 2020a). Immunization of animals with toxoids, non-toxic subunits or selected toxin domains allows for the isolation of high-affinity antibodies with immune antibody libraries. Especially well-suited for the construction of such immune antibody libraries are macaques as there V-genes are very similar to their human counterparts (Chassagne et al., 2004; Pelat et al., 2007; Pelat et al., 2009; Rülker et al., 2012; Miethe et al., 2014; Miethe et al., 2015). Alternatively, also B-cells from vaccinated humans can be used in cases where an approved vaccine is available (Wenzel et al., 2020a).

So far, neutralizing antibodies have been successfully selected against several toxins, classified as category A agents, such as botulinum toxins causing botulism from *Clostridium botulinum* (Amersdorfer et al., 2002; Conway et al., 2010; Hu et al., 2010; Miethe et al., 2014) and anthrax toxins from *Bacillus anthracis* (Pelat et al., 2007) and also against different category B agents, such as staphylococcal enterotoxin B (Larkin et al., 2010; Chen et al., 2019). We focused on toxins produced by microorganisms and did not include e.g. plant toxins like the potential biowarfare agent ricin (Pelat et al., 2009) or anti-venom, e.g. anti-snake toxin, antibodies (Laustsen et al., 2018).

One microorganism with the high-risk to be potential used as a bioweapon is the Gram-positive, anaerobic, spore-forming bacterium *Clostridium botulinum* and other *Clostridium* spp. that produce the most toxic substances known: eight different serotypes (A-H) of botulinum neurotoxin (BoNT). Five of these serotypes (A, B, E, rarely F, only one case of H) cause human botulism. Human botulism results in flaccid muscle paralysis what requires intensive hospital care and passive immunization (Arnon et al., 2001; Barash and Arnon, 2014). The most toxic substance is serotype A with a LD_50_ of about 1 ng/kg by intravenous route, about 10 ng/kg by the pulmonary route and about 1 µg/kg for the oral route (Dembek et al., 2007). BoNTs consist of a 50 kDa light chain and a 100 kDa heavy chain linked by a disulfide bound. Receptor-mediated endocytosis followed by translocation of the light chain across the membrane into the neuronal cytosol is facilitated by two functional domains (Hc and Hn) of the heavy chain. The light chain contains the catalytic domain responsible for the BoNT toxicity. Currently botulism is treated by the application of anti-botulism immunoglobulins, such equine serum or the human serum BabyBIG against type A and B. Yet the availability of BabyBIG is limited while equine serum may cause serum sickness and hypersensitivity. Here, antibody phage display technology has been used to generate toxin-neutralizing antibodies against each serotype. One single domain antibodies (VHH) was selected by phage display of a llama immunized with a cocktail of seven BoNT toxoids (A-F) (Conway et al., 2010; Shriver-Lake et al., 2017). Other VHH have been isolated from immunized alpacas and were produced as VHH-Fc fusion. These antibodies were protective against LD50 BoNT/A and showed protection over 14 days after antibody administration (Godakova et al., 2019). Furthermore, a human antibody gene library could be generated after *in vitro* immunization with BoNT/A (Hu et al., 2010). Macaque immune libraries were used to isolate neutralizing scFv against the light chains (Lc) and heavy chains (Hc) of BoNT/A, -B and -E with nM affinities against by the EU AntibotABE project (Chahboun et al., 2011; Miethe et al., 2014; Avril et al., 2015; Rasetti-Escargueil et al., 2015). These antibodies were germline-humanized and the *in vivo* protection was analyzed in non-lethal and lethal mice challenge experiments. For BoNT/A and BoNT/B neutralizing anti-Lc and anti-Hc antibody showed only limited protection, but the combination of anti-LC and anti-HC antibodies showed a synergistic effect and 100% protection when using 2.5 µg/mice (Miethe et al., 2016). Interestingly, no neutralizing antibodies against the Hc of BoNT/E were selected, but the neutralizing anti-Lc antibody alone showed full *in vivo* protection using only 2.5 ng IgG/mice (Derman et al., 2016).

Another serious infectious disease is anthrax caused by toxins of *Bacillus anthracis*, an aerobic, Gram-positive, spore-forming bacterium found in soils all over the world. *Bacillus anthracis* secretes the lethal toxin (LT) consisting of the lethal factor (LF) and the protective antigen (PA); while the edema toxin (ET) is formed by the edema factor (EF) and PA (Liu S. et al., 2014). In the pathogenesis of anthrax only LT is essential (Inglesby et al., 2002). Production and dissemination as aerosol of anthrax toxin is easily possible highlighting its potential as a bioweapon (Oncü et al., 2003). Vaccines are currently based on the subunit PA to induce the generation of neutralizing antibodies. For treatment, commercial monoclonal antibodies against PA, such as Raxibacumab, are commonly used in combination with antibiotics (Kummerfeldt, 2014). Raxibacumab was approved by the FDA in 2012 to treat inhalational anthrax. However, as PA could be mutated and modified to escape neutralizing PA antibodies while still maintaining biological activity, the use of anti-PA antibodies alone presents a security issue. An alternative and additional target presents the LF domain. Such an antibody against the LF domain is 2LF, isolated from an immune library *via* antibody phage display technology (Pelat et al., 2007). Also a synergistic effect of the combination of an anti-PA together with an anti-LF antibody is to be expected and would improve the efficacy of the therapy.

Staphylococcal enterotoxin B (SEB) from *Staphylococcus aureus* is an example for a bacterial toxin classified as category B agent, that could potentially cause foodborne illness. *Staphylococcus aureus* produces in total twenty-one types of staphylococcal enterotoxins leading to food poisoning with abdominal cramps, diarrhea and vomiting (Thomas et al., 2006; Ono et al., 2008). The most potent toxin secreted by *S. aureus* is SEB, a single 28 kDa polypeptide. As a superantigen, it stimulates T cells leading to an overproduction of cytokines, resulting in fever, hypertension and in some cases death. Phage display was employed to identify the epitope of a SEB specific monoclonal antibody using a peptide phage library (Urushibata et al., 2010) and to generate recombinant antibodies from a murine immune library (Singh et al., 2010). Additionally, synthetic human Fab libraries was used to generate human monoclonal antibodies blocking SEB binding to MHC-II (Larkin et al., 2010) and neutralizing SEB *in vitro* and *in vivo* (Chen et al., 2019). In contrast to the therapeutic use, phage display was applied to generate nanobodies against SEB from camel immune and naive libraries useful as a diagnostic tool. These nanobodies were either directly coupled with alkaline phosphatase in a sandwich-ELISA (Sun T. et al., 2020), or used for western-blot and ELISA in an indirect detection system (Zanganeh et al., 2019).

In the late 1970ies, *Clostridioides difficile* was discovered as the pathogen causing antibiotic treatment associated diarrhea (CDAD) (Bartlett et al., 1978). Only strains expressing at least Toxin B (TcdB) mostly together with Toxin A (TcdA) result in disease and typical symptoms of CDAD (Bartlett et al., 1978). Only strains expressing at least Toxin B (TcdB), mostly together with Toxin A (TcdA), result in disease and typical symptoms of CDAD (Natarajan et al., 2013). TcdA and TcdB are homologous multi-domain single-chain proteins of 308 and 270 kDa, respectively. Both toxins share the same domain architecture. The N-terminus harbors the catalytic domain, a glucosyltransferase (GTD) acting on small Rho-GTPases (Just et al., 1995; Busch et al., 1998), followed by a cysteine protease domain (CPD) catalyzing the proteolytic auto-processing and upon translocation releasing the GTD into the cytosol (Egerer et al., 2007; Reineke et al., 2007). Pore formation and translocation of the N-terminal portion of the toxin is facilitated by the translocation domain (TLD) (Genisyuerek et al., 2011). The C-terminus of TcdA and TcdB composes repetitive elements that combine long and short repeats in so called CROPs (Combined Repetitive Oligo Peptides). The CROPs are responsible for the first contact of the toxin with the target cells by interaction with carbohydrate structures on the cell surface (von Eichel-Streiber et al., 1992).

Due to increasing CDAD case numbers and epidemic outbreaks (Rupnik et al., 2009; Depestel and Aronoff, 2013), these two toxins were targets of several antibody-generation-campaigns (Babcock et al., 2006; Marozsan et al., 2012). In 2011, Hussack and coworkers were the first to report the successful generation of anti TcdA antibodies by phage display using a llama derived immune library. Four VHH antibodies (A4.2, A5.1, A20.1, and A26.8), showed potent neutralization of the cytopathic effects of TcdA on fibroblast cells in an *in vitro* assay (Hussack et al., 2011). Similarly, Yang et al. used an alpaca derived immune library to generate six and eleven unique VHH antibodies against TcdA and TcdB, respectively. The single domain antibodies showing the most potent neutralizing activity and highest affinity to non-overlapping epitopes were combined in a tetravalent bispecific antibody format designated as “ABA”. The heterotetramer ABA consists of two copies of the anti TcdB VHH E3 as well as the one copy of each of the anti TcdA VHHs AH3 and AA6 in the following order: AH3/E3/E3/AA6. ABA was shown to bind to TcdA and TcdB simultaneously and one single injection of ABA was enough to reverse fulminant CDAD in mice. In another antibody generation campaign, the naïve scFv libraries HAL9/10 (Kügler et al., 2015) were used to isolate the first fully human, phage display derived antibodies against TcdB. Using various TcdB fragments for the panning process, partially combined with preincubation steps, 31 unique antibodies were generated directed against a broad range of epitopes. The antibodies were tested in an *in vitro* neutralization assay using Vero cells. Interestingly, the two most potent antibodies bound to the same, formerly unknown, epitope within the glycosyltransferase domain of TcdB (Fühner et al., 2018).

Diphtheria toxin (DT) is a typical A-B-toxin with an A subunit (catalytic domain) and a B subunit that contains the cell binding domain and the translocation domain. DT is produced by a corynephage β infected pathogenic *Corynebacterium* stain, mainly *Corynebacterium diphtheriae* (Murphy, 1996). To date, the only approved therapeutic is a serum from horses immunized with diphtheria toxin, as invented by Emil von Behring and Shibasaburo Kitasato over 100 years ago (von Behring and Kitasato, 1890). Antibodies were generated by phage display from immune libraries of boost vaccinated donors. Neutralizing antibodies against all three domains of DT were selected. The best antibodies showed an *in vitro* neutralizing antibody with a relative potency of 454 IU/mg at 4xMCDt (minimal cytopathic dose). When using higher toxin concentration, the neutralization efficacy was highly reduced, but when using two or three antibodies in combinations the neutralization was restored. An *in vivo* potency of 79.4 IU/mg was achieved in an intradermal challenge assay (Wenzel et al., 2020a).

An Overview of recombinant antibodies against toxins are given in **Table 4**.

**Table 4:** Recombinant antibodies derived by phage display against toxins.

Toxin	Species	Target	library type	Antibody Format	Antibody Origin	Application	Reference
Adenylate cyclase toxin (ACT)	*Bordetella pertussis*	C-terminal repeat-in-toxin (RTX) domain of ACT	immune	scFv	mice	ELISA, *in vitro* inhibition	(Wang X. et al., 2015)
Alpha-toxin	*Clostridium perfringens*	type A alpha-toxin	semi-synthetic	scFv	human	ELISA, *in vitro* inhibition, *in vivo* protection	(Wang D. et al., 2017)
Anthrax toxin	*Bacillus anthracis*	lethal factor (LF)	immune	scFv	macaque	ELISA, *in vitro* toxin neutralization, *in vivo* protection	(Pelat et al., 2007)
Anthrax toxin	*Bacillus anthracis*	protective antigen (PA)	naïve	scFv	human	*in vitro* toxin neutralization, *in vivo* protection	(Mazumdar, 2009)
Anthrax toxin	*Bacillus anthracis*	protective antigen (PA)	immune	VHH	camel	ELISA	(Shali et al., 2018)
Anthrax toxin	*Bacillus anthracis*	lethal factor (LF)	immune	VHH, bispecific VHH	alpaca	ELISA, WB, *in vivo* protection	(Vrentas et al., 2016)
Anthrax toxin	*Bacillus anthracis*	protective antigen (PA)	immune	VHH, bispecific VHH	alpaca	ELISA, *in vitro inhibition*, *in vivo* protection	(Moayeri et al., 2015)
Botulinum Neurotoxin	*Clostridium botulinum*	serotype A - light chain	immune	scFv	macaque	ELISA, *in vitro* toxin inhibition	(Chahboun et al., 2011)
Botulinum Neurotoxin	*Clostridium botulinum*	serotype A - light chain	immune	scFv,scFv-Fc	macaque	ELISA, WB, *in vitro* toxin inhibition, ex vivo toxin neutralization	(Miethe et al., 2014)
Botulinum Neurotoxin	*Clostridium botulinum*	serotype A - light chain	immune	VHH	camel	ELISA, WB, *in vitro* neutralization	(Thanongsaksrikul et al., 2010)
Botulinum Neurotoxin	*Clostridium botulinum*	serotype A - heavy chain	immune	scFv	murine	ELISA, *ex vivo* toxin neutralization	(Amersdorfer et al., 1997)
Botulinum Neurotoxin	*Clostridium botulinum*	serotype A - heavy chain	immune	scFv	human	ELISA, *ex vivo* toxin neutralization	(Amersdorfer et al., 2002)
Botulinum Neurotoxin	*Clostridium botulinum*	serotype A - heavy chain	naïve	scFv	human	ELISA, *ex vivo* toxin neutralization	(Amersdorfer et al., 2002)
Botulinum Neurotoxin	*Clostridium botulinum*	serotype A - heavy chain	immune	scFv	macaque	ELISA, *ex vivo* toxin neutralization	(Avril et al., 2015)
Botulinum Neurotoxin	*Clostridium botulinum*	serotype A + B	immune	scFv-Fc, IgG	macaque	ELISA, *in vivo* mouse paralysis assay, *in vivo* protection	(Miethe et al., 2016)
Botulinum Neurotoxin	*Clostridium botulinum*	serotype A	immune	VHH, VHH-Fc	alpaca	*in vivo* protection	(Godakova et al., 2019)
Botulinum Neurotoxin	*Clostridium botulinum*	serotype B - light chain/heavy chain	immune	scFv,scFv-Fc	macaque	ELISA, *in vitro* toxin inhibition, ex vivo toxin neutralization, *in vivo* protection	(Rasetti-Escargueil et al., 2015)
Botulinum Neurotoxin	*Clostridium botulinum*	serotype E - light chain	immune	scFv,scFv-Fc	macaque	ELISA, *in vitro* toxin inhibition, ex vivo toxin neutralization, *in vivo* protection	(Miethe et al., 2015)
Botulinum Neurotoxin	*Clostridium botulinum*	serotype E - light chain	immune	scFv-Fc, IgG	macaque	ELISA, *in vivo* mouse paralysis assay, *in vivo* protection	(Derman et al., 2016)
Botulinum Neurotoxin	*Clostridium botulinum*	serotype E - heavy chain	immune	VHH	dromedary	ELISA, *in vivo* protection	(Bakherad et al., 2013)
Botulinum Neurotoxin	*Clostridium botulinum*	serotype A/B/C/D/E/F	immune	VHH	llama	ELISA, *in vitro* toxin inhibition	(Conway et al., 2010; Shriver-Lake et al., 2017)
*C. difficile* toxin	*Clostridioides difficile*	TcdA	immune	VHH	llama	ELISA, WB, *in vitro* neutralization	(Hussack et al., 2011)
*C. difficile* toxin	*Clostridioides difficile*	TcdA, TcdB	immune	VHH, bispecific VHH	alpaca	ELISA, *in vitro* toxin inhibition, *in vivo* protection	(Yang et al., 2014)
*C. difficile* toxin	*Clostridioides difficile*	TcdB	naïve	scFv-Fc	human	dot blot, *in vitro* neutralization	(Chung et al., 2018)
*C. difficile* toxin	*Clostridioides difficile*	TcdB	naïve	scFv, scFv-Fc	human	ELiSA, *in vitro* neutralization	(Fühner et al., 2018)
*C. difficile* toxin	*Clostridium difficile*	binary CDT toxin	immune	VHH, VHH-Fc	llama	ELISA, *in vitro* toxin inhibition, IF	(Unger et al., 2015)
Cry toxin	*Bacillus thuringiensis*	Cry1Aa, Cry1Ab, Cry1Ac, Cry1B, Cry1C, Cry1F	immune	scFv	mice	ELISA, sandwich ELISA	(Dong et al., 2018)
Cry toxin	*Bacillus thuringiensis*	Cry1E	immune	dAb	human	ELISA, *in vitro* inhibition	(Xu et al., 2017a)
Cry toxin	*Bacillus thuringiensis*	Cry1F	immune	scFv	rabbit	ELISA	(Xu et al., 2017b)
Cry toxin	*Bacillus thuringiensis*	Cry3A	immune	VHH	llama	ELISA	(Zúñiga-Navarrete et al., 2015)
Cry toxin	*Bacillus thuringiensis*	Cry1Fa	immune	VHH	camel	sandwich ELISA	(Wang P. et al., 2014)
Cry toxin	*Bacillus thuringiensis*	Cry1C d-endotoxins	semi-synthetic	scFv	human	ELISA, *in vitro* toxin inhibition	(Wang Y. et al., 2012)
Cry toxin	*Bacillus thuringiensis*	Cry1B toxin	immune	VHH	camel	ELISA	(Zhong et al., 2018)
CyaA-hemolysin	*Bordetella pertussis*	CyaA-Hly	semi-synthetic	VHH	humanizedcamel	ELISA, WB, *in vitro* neutralization	(Malik et al., 2016)
Diphtheria toxin	*Corynebacterium diphtheriae*	DT	immune	scFv, scFv-Fc, IgG	human	ELISA, *in vitro* neutralization, in *vivo* neutralization	(Wenzel et al., 2020a)
Enterotoxin B	*Escherichia coli*	EtxB	semi-synthetic	scFv	human	ELISA, *in vitro* toxin inhibition	(Chung et al., 2008)
Epsilon toxin	*Clostridium perfingens*	Epsilon toxoid	semi synthetic	scFv, dAb	human	ELISA	(Alibeiki et al., 2020)
Mycotoxin	*Fusarium verticillioides*	fumonisin B1	semi-synthetic	scFv	human	–	(Lauer et al., 2005)
Mycotoxin	*Fusarium* spp.	trichothecenes HT-2	immune	Fab	mice	TR-FRET Assay	(Arola et al., 2016)
Mycotoxin	*Aspergillus flavus*	aflatoxin B1	immune	VHH	alpaca	ELISA	(He et al., 2014)
Mycotoxin	*Fusarium* spp.	deoxynivalenol	synthetic	scFv	human	ELISA,	(Leivo et al., 2020)
Microcystin	*Microcystis aeruginosa*	ADDA	semi-synthetic	scFv	human	ELISA	(McElhiney et al., 2000)
Microcystin	*Microcystis aeruginosa*	microcystin-LR	immune	scFv	rabbit	ELISA	(Xu et al., 2019)
Microcystin	*Microcystis aeruginosa*	microcystin-LR	immune	VHH	alpaca	ELISA, inhibition ELISA	(Xu et al., 2018)
Mycolactone	*Mycobacterium ulcerans*	lipidic toxin A	naïve	scFv, scFv-Fc	human	ELISA, flow cytometry,	(Naranjo et al., 2019)
Nodularin	*Nodularia spumigena*	NOD	semi-synthetic	scFv, scFv-alkaline phosphatase	human	competition assay	(Akter et al., 2017)
*Pseudomonas aeruginosa* exotoxin A	*Pseudomonas aeruginosa*	ETA	naïve	scFv	human	ELISA, WB, *in vitro* inhibition	(Santajit et al., 2019)
*Pseudomonas aeruginosa* exotoxin A	*Pseudomonas aeruginosa*	ExoA-DI	semi-synthetic	scFv	human	ELISA, WB	(Shadman et al., 2021)
Salmonella Typhi Hemolysin E	*Salmonella* Typhi	hemolysin E antigen	naïve	scFv	human	ELISA, WB	(Lim et al., 2016)
Shiga toxin	*E.coli* (STEC)	Stx2	semi-synthetic	Fab	human	ELISA, WB, *in vitro* neutralization	(Luz et al., 2015)
Shiga toxin	*E.coli* (EHEC)	Stx1, Stx2	naïve	scFv	human	ELISA, FACS, *in vitro* toxin neutralization	(Neri et al., 2011)
Shiga toxin	*E.coli* (STEC)	Stx1, Stx2	immune	VHH	alpaca	ELISA, *in vitro* toxin inhibition, *in vivo* protection	(Tremblay et al., 2013)
Shiga toxin	*E.coli* (STEC)	Stx2e	immune	VHH	llama	ELISA, *in vitro* neutralization	(Lo A. W. H. et al., 2014)
*Staphylococcus* enterotoxin B	*Staphylococcus aureus*	SEB	immune	scFv	mice	ELISA	(Singh et al., 2010)
*Staphylococcus* enterotoxin B	*Staphylococcus aureus*	SEB	synthetic	Fab	human	ELISA, WB, *in vitro* toxin inhibition	(Larkin et al., 2010)
*Staphylococcus* enterotoxin B	*Staphylococcus aureus*	SEB	synthetic	Fab, IgG	human	ELISA, *in vivo* protection	(Chen et al., 2019)
*Staphylococcus* enterotoxin B	*Staphylococcus aureus*	SEB	naïve	VHH, VHH-alkaline phosphatase fusion	camel	ELISA, chemiluminescent immunoassay	(Sun T. et al., 2020)
*Staphylococcus* enterotoxin B	*Staphylococcus aureus*	SEB	immune	VHH	camel	ELISA	(Zanganeh et al., 2019)
Tetanus neurotoxin	*Clostridium tetani*	tetantus toxoid	immune	Fab	macaque	ELISA	(Chassagne et al., 2004)
Tetanus neurotoxin	*Clostridium tetani*	tetantus toxoid	naïve	scFv	human	ELISA, *in vitro* toxin inhibition	(Indrawattana et al., 2010)
Tetanus neurotoxin	*Clostridium tetani*	TeNT heavy chain	immune	scFv, IgG	human	ELISA, immunofluorescence, *in vitro* inhibition	(Wang H. et al., 2016)
Tetanus neurotoxin	*Clostridium tetani*	tetanus neurotoxin (TeNT)	immune	scFv	human	*in vitro* inhibition	(Khalili et al., 2015)
Tetanus neurotoxin	*Clostridium tetani*	tetantus toxoid	naïve	Fab	human	ELISA, *in vitro* neutralization	(Neelakantam et al., 2014)
Tetanus neurotoxin	*Clostridium tetani*	TeNT	Immune	ScFv, IgG	human	ELISA	(Sadreddini et al., 2015)
Toxic shock syndrome toxin-1	*Staphylococcus aureus*	TSST-1	naïve	scFv	human	T-cell activation assay, interleukine gene expression	(Rukkawattanakul et al., 2017)
Vacuolating cytotoxin A	*Helicobacter pylori*	p55 domain of VacA	semi-synthetic	scFv	human	ELISA, WB, FACS	(Fahimi et al., 2018)
Vibrio parahaemolyticus hemolysin	*Vibrio parahaemolyticus*	TLH	immune	scFv	mice	ELISA, FACS, *in vitro* neutralization	(Wang R. et al., 2012)
Vibrio vulnificus toxin	*Vibrio vulnificus*	VvRtxA	semi-synthetic	scFv	human	ELISA, *in vitro* toxin inhibition, IF	(Hsu et al., 2011)

ELISA, enzyme linked immunosorbent assays; MS, mass spectrometry; WB, western blot.

## Conclusion

A large number of recombinant antibodies for application in diagnostics and therapy were already generated by phage display against viral, bacterial and eukaryotic pathogens as well as toxins. Antibody phage display allows the generation of antibodies originating from several species, including human, camel, llama, alpaca, chimpanzee, macaque, pig, mice, chicken or shark. These antibodies are derived from two types of library sources: immune or universal libraries. Immune libraries are preferred when convalescent patients or immunized animals/human donors are available. This approach offers the advantage to directly isolate affinity matured antibodies. Universal antibody gene libraries offer an alternative if immunization is not possible, ethically not feasible or patient samples are not available.

The COVID-19 pandemic should teach us to be prepared for the next pandemic, which beyond any doubt will come. We need permanent preparedness platforms which will react on novel pathogens arising worldwide and provide antibodies for diagnostics and as potential therapeutic lead candidates. This preparedness platforms should also have a budget for immediate start of GMP production and clinical phase I/II studies. In the most cases, new pathogens may have only a local impact, but this cannot be assured. Also these antibodies are always valuable tools for research and diagnostics of the pathogens.

We do not build a fire department when the house is already burning, we need to establish it in advance and maintain this service also in times without emergency. We have to think in the same way about infectious diseases and potential pandemics.

## Author Contributions

All authors listed have made a substantial, direct, and intellectual contribution to the work, and approved it for publication.

## Funding

We acknowledge funding from the European Community’s Seventh Framework Program (FP7/2007-2013) under agreement no. 241832 granted to the AntiBotABE project (http://www.antibotabe.com) and funding received from the European Union’s Horizon 2020 research and innovation programme under grant agreement No 101003650, funding from Federal State of Lower Saxony, Niedersächsisches Vorab (VWZN2889) and financial support of MWK Niedersachsen (14-76103-184 CORONA-2/20). We thank CNPq for supporting the scholarship of GMSGM. This review is an updated and revised version of a formerly published review (Kuhn et al., 2016).

## Conflict of Interest

EW, GR, and SD are shareholders of Abcalis GmbH. AF, TS, SD, and MH are shareholders of YUMAB GmbH.

The remaining authors declare that the research was conducted in the absence of any commercial or financial relationships that could be construed as a potential conflict of interest.
